# Supplementary material for: Ultra-narrow donor-acceptor nanoribbons
Source: Nat Commun. 2026 Apr 23;17:3492. doi: 10.1038/s41467-026-71660-0 (PMC13106672; doi:10.1038/s41467-026-71660-0)
Supplement: Supplementary file 1 — Supplementary Information [file 41467_2026_71660_MOESM1_ESM.pdf]

Supplementary Information

Ultra-narrow donor-acceptor nanoribbons

James Lawrence,[1,2]* Luka Đorđević,[3,†] Fabienne Bachtiger[1], Harry Pinfold,[1] Marc Walker,[4] Jiong Lu,[2,5] Gabriele C. Sosso,[1] Davide Bonifazi,[3,6] Giovanni Costantini[1,7,8]*

[1]Department of Chemistry, University of Warwick, Coventry CV4 7AL, UK

[2]Department of Chemistry, National University of Singapore, 117543, Singapore

[3]School of Chemistry, Cardiff University, Cardiff CF10 3AT, UK

[4]Department of Physics, University of Warwick, Coventry CV4 7AL, UK

[5]Institute for Functional Intelligent Materials, National University of Singapore, 117544, Singapore

[6]Department of Organic Chemistry, University of Vienna, 1090 Vienna, Austria

[7]School of Chemistry, University of Birmingham, Birmingham B15 2TT, UK

[8]School of Physics and Astronomy, University of Birmingham, Birmingham B15 2TT, UK

[†]Present address: Department of Chemical Sciences, University of Padova, Padova 35131, Italy

Table of contents

1. Hybridisation of frontier molecular orbital levels in D-A couplings 3
2. Precursor molecules and electronic character of short nanoribbons 4
3. STM images and XP spectra of Br_2PXX/Au(111) as a function of annealing 5
4. Possible precursor coupling pathways 6
5. Contaminant derivatives of Br_2PXX 7
6. Coupling of misaligned reactive sites 8
7. BR-STM images of PXX nanoribbons of different length 9
8. BR-STM images of Br_2PXX and mixed Br_2PXX/VO3 samples after annealing to 473 K 9
9. Possible reaction pathways for the nanoribbon synthesis 10
10. STM images of self-assembled VO3 11
11. STM images of VO3/Au(111) as a function of annealing 12
12. Self-assembly of AO nanoribbons 12

13. Defective coupling of AO nanoribbons 13
14. Positions of dI/dV spectra recorded on short, pure nanoribbons 14
15. Comparison of dI/dV images recorded with *s*- and *p*-wave tips 15
16. Energy shift & emptying of the highest occupied state for longer PXX nanoribbons 15
17. STS of a 21-unit long PXX nanoribbon 17
18. Statistical analysis of D/A combinations in mixed dimers 17
19. Coupling geometries of DD, AA and DA junctions 19
20. Positions of dI/dV spectra recorded on mixed D-A nanoribbons 19
21. Simple LCMO derivation of HOMO/LUMO energies of mixed D-A trimers 20
22. Variability of dI/dV spectra recorded on PXX-AO nanoribbon dimers 24
23. Effect of environment on dI/dV resonances of Br_2PXX molecules 25
24. A comparison between images recorded with two different CO tips 26
25. Differentiating between the functional groups with bond-resolving techniques 27
26. DFT calculations - Influence of the exchange-correlation functional 27
27. Synthetic method 28

1. Hybridisation of frontier molecular orbital levels in D-A couplings

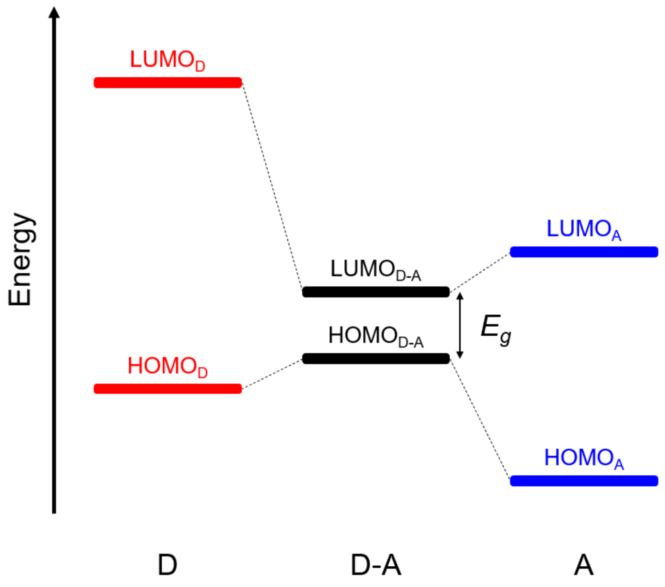

Figure S1. Schematic representation of the hybridisation of the frontier molecular orbital levels of a DA unit.

2. Precursor molecules and electronic character of short nanoribbons

The chemical structure of the electron donor *peri*-xanthenoxanthene (PXX) and the electron acceptor anthanthrone (AO), used for synthesising the ultra-narrow nanoribbons, are shown in Fig. S2(a), along with the structure of their all-carbon analogue, anthanthrene (AA) and its hypothetical brominated form, Br_2AA.

As the length of the nanoribbons increases, the donor or acceptor strength of PXX and AO correspondingly increases due to a progressive narrowing of the band gap. This trend is confirmed by gas-phase DFT calculations of the HOMO and LUMO energies for short nanoribbons of PXX, AO and AA, presented in Fig. S2(b). It is important to note that while DFT captures relative orbital energies and gaps, the absolute positions of HOMO and LUMO are not physically meaningful, as Kohn-Sham orbitals are auxiliary constructs which absolute energies depend on both the choice of the exchange-correlation functional and the choice of the (arbitrary) energy zero. As these calculations were performed for isolated oligomers in the gas phase, the natural and consistent choice here was to reference the energy levels to the vacuum level. We further note that brominated analogues of AA are likely too reactive for practical use as on-surface precursors, owing to their central zigzag-edge carbon atoms, which are prone to oxidation under ambient conditions. Similar instability has been reported for chiral nanoribbons containing zigzag edges with a similar length.[1,2] In contrast, less reactive dibromopyrene precursors have been employed in previous studies to synthesise comparable all-carbon nanoribbons.[3]

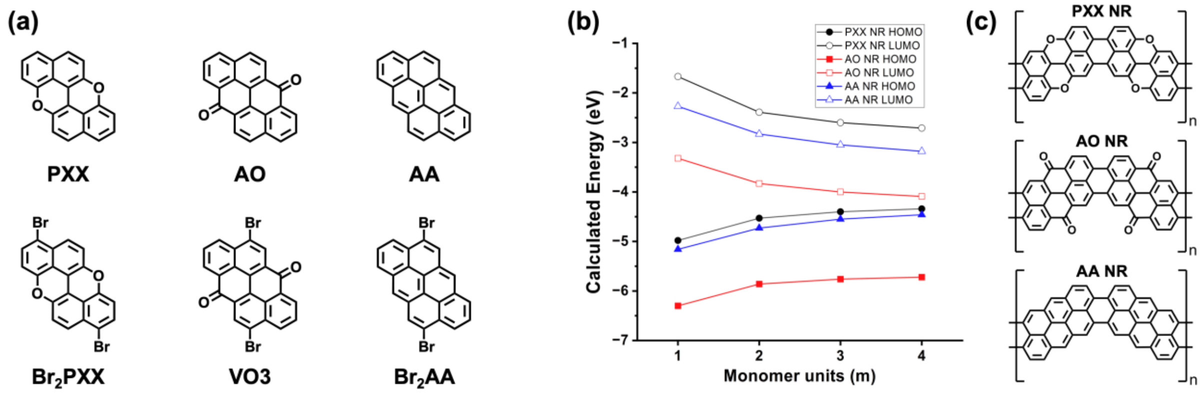

Figure S2. (a) Chemical structures of PXX, Br_2PXX, AO, VO3, AA and Br_2AA. (b) DFT-calculated HOMO and LUMO energies of nanoribbons of PXX, AO and AA of increasing length. The donor and acceptor characters of PXX and AO, respectively, are clear from their relative HOMO and LUMO energies. (c) Structures of the different nanoribbons formed by the molecules in (a). The repeat unit shown here contains two monomer units.

3. STM images and XP spectra of Br_2PXX/Au(111) as a function of annealing

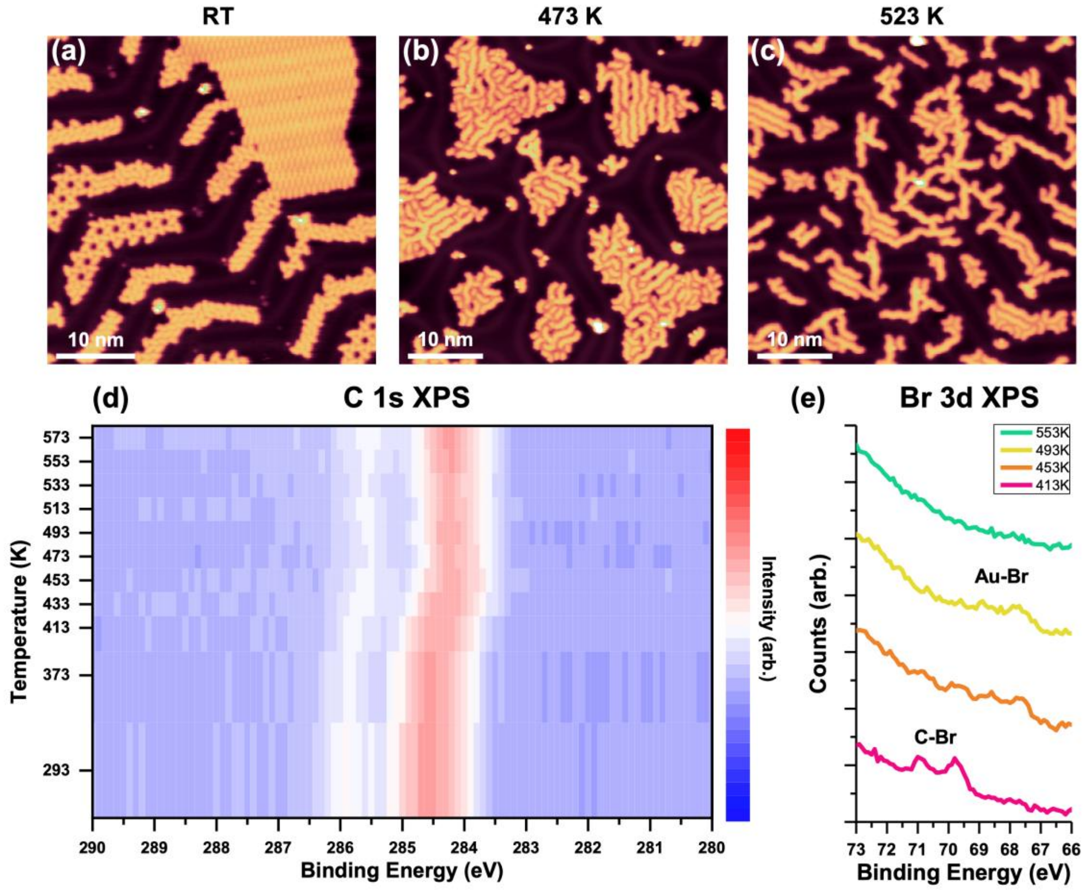

Figure S3. (a-c) Large-scale STM images (recorded at 7 K) of a sample of Br_2PXX on Au(111) at different annealing temperatures. (d) C 1*s* X-ray photoemission (XP) spectra showing the change in carbon environment that is evident upon nanoribbon formation above 413 K. The less intense, higher binding energy peak corresponds to the carbon atoms bonded to oxygen atoms in the molecules/ribbons. (e) Br 3*d* XP spectra of a Br_2PXX/Au(111) sample after successive annealing steps. These spectra demonstrate that the debromination of Br_2PXX has started between 413 K and 453 K (forming a chemisorbed Au-Br species with a different binding energy) and that the bromine atoms have fully desorbed after annealing to 553 K. Imaging parameters: (a) I_T = 110 pA, V_b = +1.13 V; (b) I_T = 190 pA, V_b = +1.06 V; (c) I_T = 170 pA, V_b = +1.06 V.

Whilst STM can give an insight into the structure of species at the nanoscale, it does not necessarily provide chemical information. As such, we have recorded C 1*s* XP spectra at various annealing steps, as presented in Fig. S3(d), in order to determine changes to the chemical environment of the carbon atoms in the nanoribbons and thus fully prove their successful formation. For all of the C 1*s* spectra, a clearly separate peak at a higher binding energy (285.9 eV) is observed, corresponding to the carbon atoms that are bound to the oxygen atoms. A gradual transition to lower binding energies is observed between 413 K and 453 K and is interpreted as the result of the transition from precursor to nanoribbon in a similar manner to that seen in other studies.[4,5] We cannot rule out that part of this transition is related to the formation of intermediate species, such as organometallic C–Au–bonded structures. However, if such intermediates occur, they did not appear as a distinct reaction step that can be clearly identified in corresponding STM experiments. We also do not observe a pronounced shift in the C 1*s* binding energy upon bromine desorption that would be expected from a significant

work-function change, although a subtle upshift may be present at temperatures above 473 K in Fig. S3(d).

Fig. S3(e) shows the Br 3*d* binding energy (BE) region for four different annealing steps, with each spectrum recorded after the sample had been cooled down to room temperature. While the intensity of the signal is rather weak, it is clear that two distinct peaks can be observed at the lowest annealing temperature, corresponding to the Br $3d_{5/2}$ and $3d_{3/2}$ components of bromine atoms bound to C in the Br_2PXX precursor molecule. Starting at the annealing temperature of 453 K and, more distinctly, at 493 K, a new pair of Br 3*d* peaks is observed, shifted to a lower BE. This corresponds to bromine atoms that are bound directly to the metal substrate, proving the debromination of the molecules. This fits with the observation of self-assembled islands containing both nanoribbons and Br atoms in the STM measurements. The final annealing step to 553 K leads to the desorption of the bromine atoms, as shown by the absence of any signal in the corresponding spectrum. The precise temperature at which this occurs is coverage-dependent; the slightly lower coverage sample shown in Fig. 2(b) had almost no apparent Br atoms remaining after a 523 K anneal, whereas the higher coverage sample in Fig. S3(c) still had a few remaining.

4. Possible precursor coupling pathways

During nanoribbon synthesis, no intermediates were detected by STM at annealing temperatures below those required for nanoribbon formation, suggesting that Ullmann coupling and dehydrogenation likely occur simultaneously. The lack of any covalently bonded polymeric intermediates (as seen in many other nanoribbon formation studies) may be related to the twisted geometry that such a structure would require due to the steric clashing of the C-H groups between the units, perhaps disfavouring its formation. This is different from precursors used in other nanoribbon formation studies, such as the commonly used 10,10-dibromo-9,9 bianthracene (DBBA), which are non-planar and often do not require a significant change in their adsorption structure when undergoing the initial Ullmann coupling step.[6,7] Only a few examples of a similar coupling to form nanoribbons from planar precursors can be found in the literature.[3,5,8,9] A clearly defined organometallic intermediate step[10] (prior to the nanoribbon formation) was also generally not observed. However, after annealing to the higher temperature of 473 K, a mixture of species was found for both precursors used in this study (Br_2PXX and VO3), with a few examples of molecules that appeared to have a bright feature adjacent to the carbon that formerly hosted a C-Br group (Fig. S8). These features could tentatively be associated with Au atoms bound to the debrominated monomers. However, these species were never isolated with a lower annealing temperature and always appeared alongside fully polymerised/dehydrogenated products.

These results recall the observations reported for the synthesis of 5-armchair graphene nanoribbons from dibromoperylene, a flat dibrominated precursor that bears some similarities with Br_2PXX.[5] Also in that study a mixture of intact molecules, organometallic chains, and fully fused nanoribbons was found at a similar annealing temperature, together with several examples of kinked nanoribbons connected by 5-membered rings (identified as kinetic by-products). Supported by DFT calculations, Berdonces-Layunta *et al.* proposed a concerted rather than stepwise synthesis of the nanoribbons, with C-C bond formation and cyclodehydrogenation occurring in one step due to the activation of the C-H bonds in the presence of the adjacent radicals resulting from debromination (Fig. S9).[5] We suggest that this is most likely also the case for the nanoribbons presented in this study. It should be noted that this mechanism is the on-surface equivalent of the single-step ladderization synthesis of fully conjugated ladder polymers, a method that is not widely used in solution due to the limited availability of multifunctional monomers and their general poor solubility.[11]

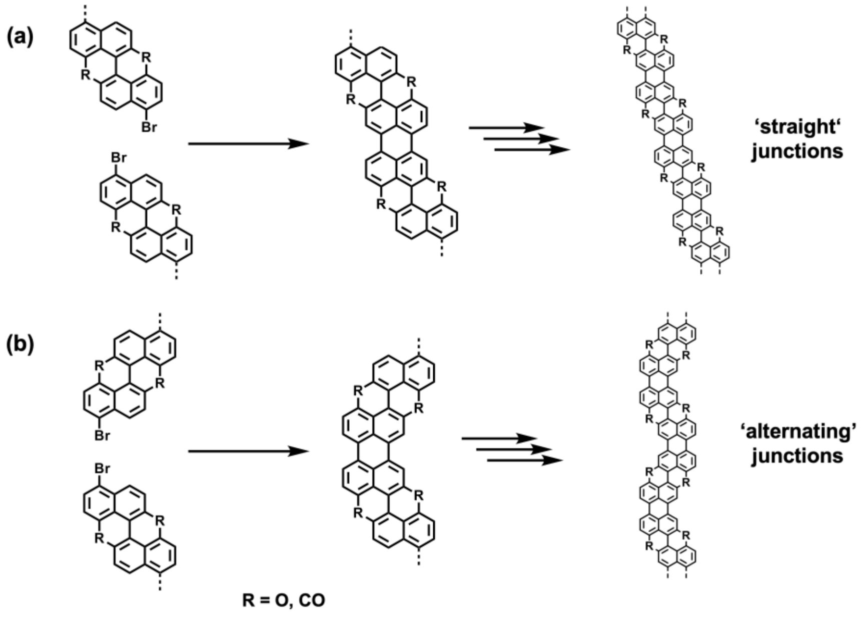

Figure S4. Two possible formation pathways for the nanoribbons. (a) The cross-coupling reaction between units of the same pro-chirality leads to 'straight' junctions. (b) The homo-coupling reaction between two units with an opposite pro-chirality results in 'alternating' junctions.

5. Contaminant derivatives of Br_2PXX

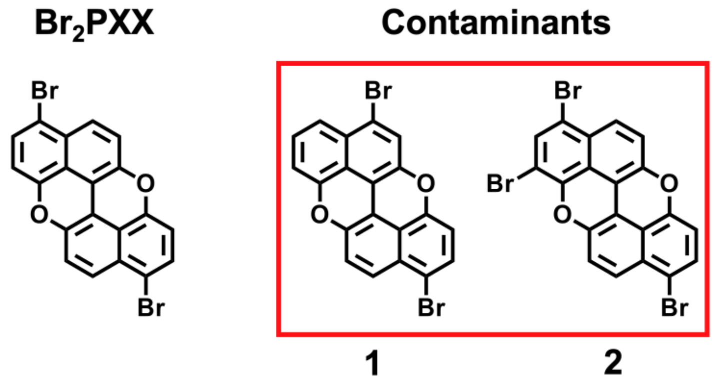

Figure S5. Structure of Br_2PXX, alongside the two main contaminant derivatives that were observed when subliming the molecule onto the Au(111) surface.

Two main contaminant derivatives of Br_2PXX were imaged with BR-STM and reported in our previous study on the self-assembly of Br_2PXX.[12] It is likely that these contaminants contribute to the formation of the defective nanoribbons and branched chains that are occasionally observed on the surface. In particular, the presence of molecules with a bromine on the wrong end position (contaminant 1) can lead to the formation of 'straight' ribbon junctions rather than the regular 'alternating' structures (see Fig. S4), while contaminants with three bromine atoms (contaminant 2) can cause branching of the ribbons.

6. Coupling of misaligned reactive sites

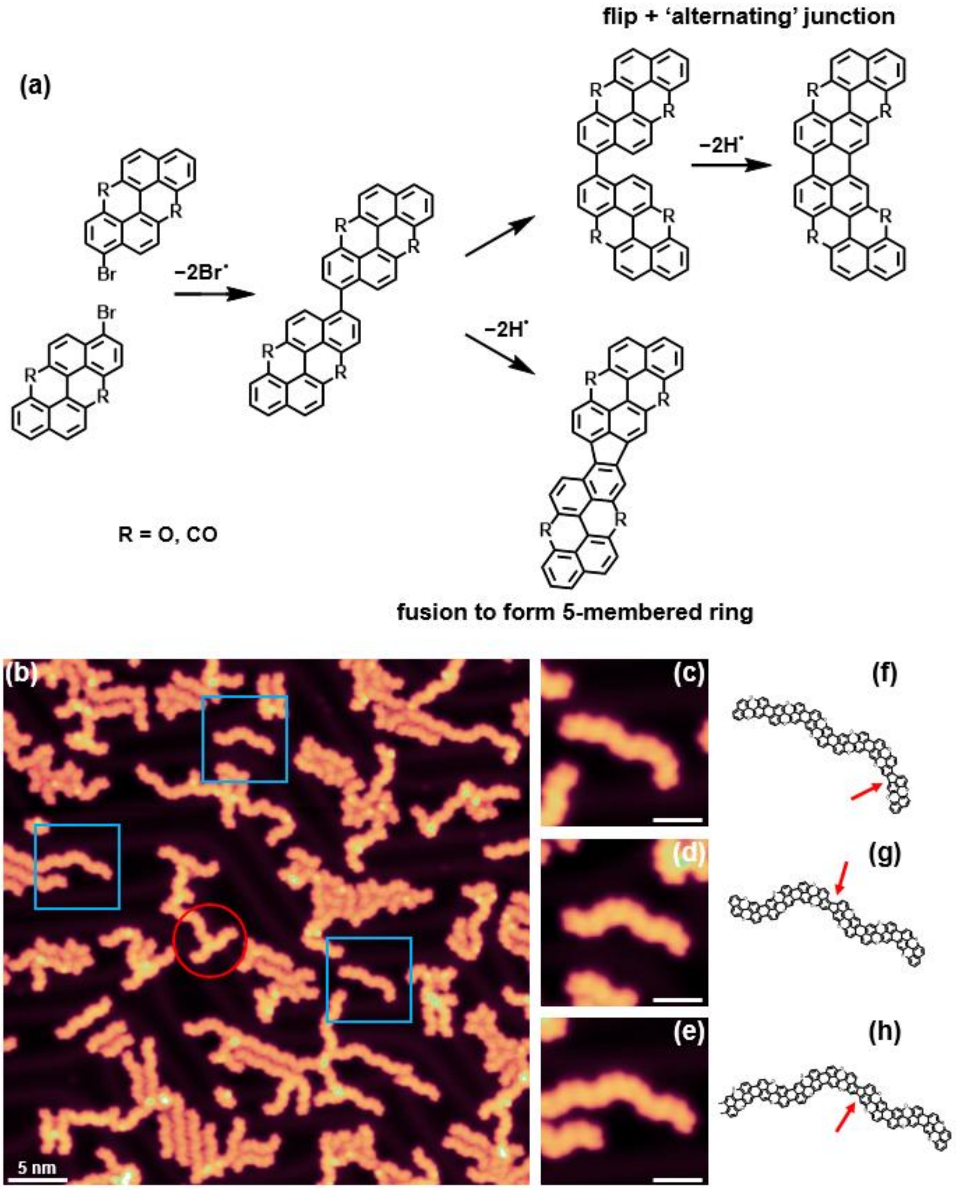

Figure S6. (a) Two possible reaction pathways for the situation in which the reactive nanoribbon termini have bonded at their former C-Br positions but are not aligned in a way that would form the conventional nanoribbon junctions shown in Fig. S4. The resulting intermediate molecule can either rotate around the newly formed C-C bond, doubly dehydrogenate and form a second C-C bond, ending in a regular 'alternating' junction (top path); or instead immediately doubly dehydrogenate and form a second C-C bond, resulting in the formation of a 5-membered ring and a kink along the nanoribbon (bottom path). By involving a flip – and thus the detachment of part of the nanoribbon from the metallic substrate – the first option is expected to be energetically unfavourable, especially for longer nanoribbons. Note: the reactions are represented here as two-step processes, with the C-C bond formed first and the double dehydrogenation occurring later. This might however not be the case in reality and could well happen in a single step, similar to what is expected for regular nanoribbon junctions (see main text and Fig. S9). (b) STM image of PXX nanoribbons on Au(111) after annealing to 573 K. Many of the nanoribbons have kinks that indicate that they have fused by forming 5-membered rings. The red circle highlights a branched nanoribbon. (c)-(e) Crops of the image in (a) to zoom in on three examples of nanoribbons with 5-membered rings. Scale bars are 2 nm. (f)-(h) Proposed chemical structures of the nanoribbons in (c)-(e), with red arrows indicating the position of the 5-membered rings. Imaging parameters for (b)-(e): I_T = 520 pA, V_b = –1.33 V.

7. BR-STM images of PXX nanoribbons of different length

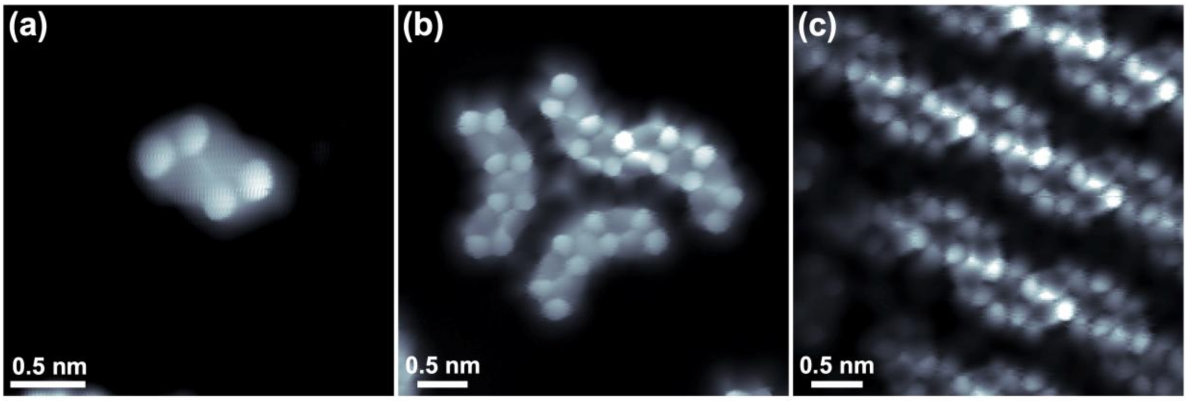

Figure S7. Constant-height CO tip BR-STM images of PXX nanoribbons of increasing length, recorded at a low bias voltage (V_b = +40 mV): (a) shows a PXX monomer, (b) two dimers and a trimer and (c) much longer nanoribbons. The images show a clear change in contrast caused by the upshift of the highest-occupied state closer to the Fermi level with increasing ribbon length (Fig. S16). There is a noticeable contrast change even when comparing the dimers to the trimer in (b), with the centre of the trimer appearing distinctly brighter. Despite these differences, the rings can mostly still be discerned within longer ribbons in BR-STM, with strong changes in contrast over the central oxygen-containing rings (as seen with the precursor in a previous study).[12]

8. BR-STM images of Br_2PXX and mixed Br_2PXX/VO3 samples after annealing to 473 K

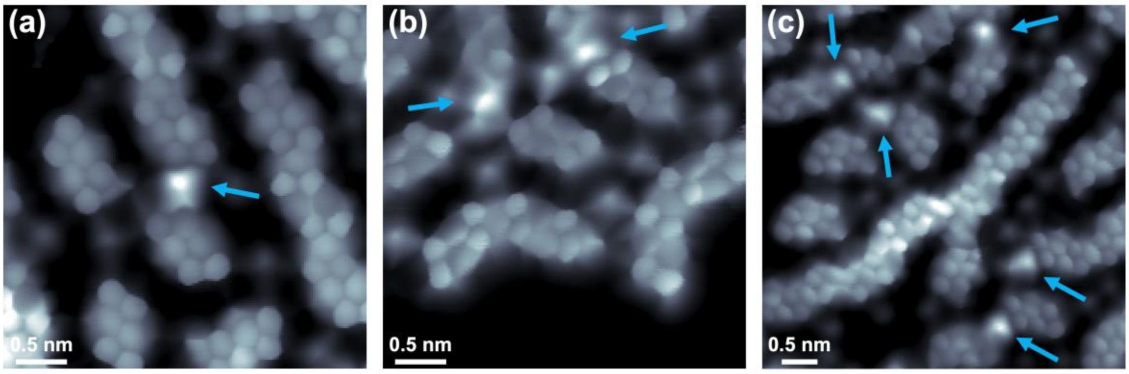

Figure S8. Constant height BR-STM images (V_b = +40 mV) of islands composed of Br atoms and nanoribbons, obtained after annealing to 473 K. (a) and (c) were recorded on a mixed Br_2PXX/VO3 sample, whereas (b) was recorded on a pure Br_2PXX sample. The blue arrows indicate the positions of possible Au atoms in organometallic intermediates. The potential Au atoms appear as bright features, close to the expected positions for the carbon radicals that are formed upon debromination. Some of these features are shared by neighbouring molecules, but many are not.

9. Possible reaction pathways for the nanoribbon synthesis

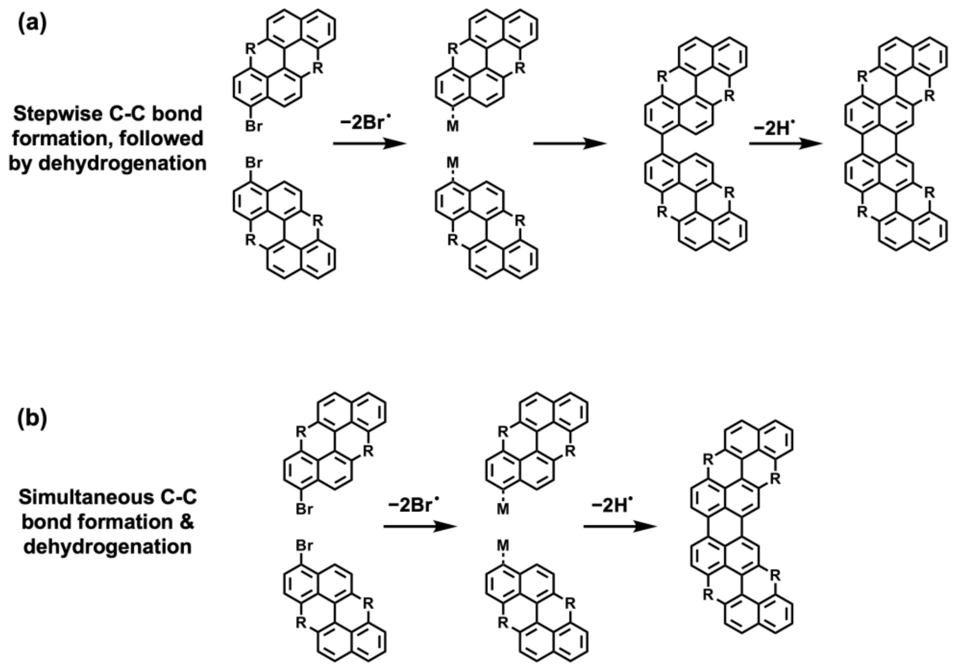

Figure S9. The two possible reaction pathways for the nanoribbon synthesis. (a) Stepwise C-C bond formation, followed by dehydrogenation. Two metal atoms are shown, but a single one could also be shared between the units. (b) Concerted C-C bond formation and cyclodehydrogenation occurring due to the activation of the C-H bonds in the presence of the adjacent radicals resulting from debromination. This latter mechanism is suggested to occur for the PXX and AO nanoribbons in this study.

10. STM images of self-assembled VO3

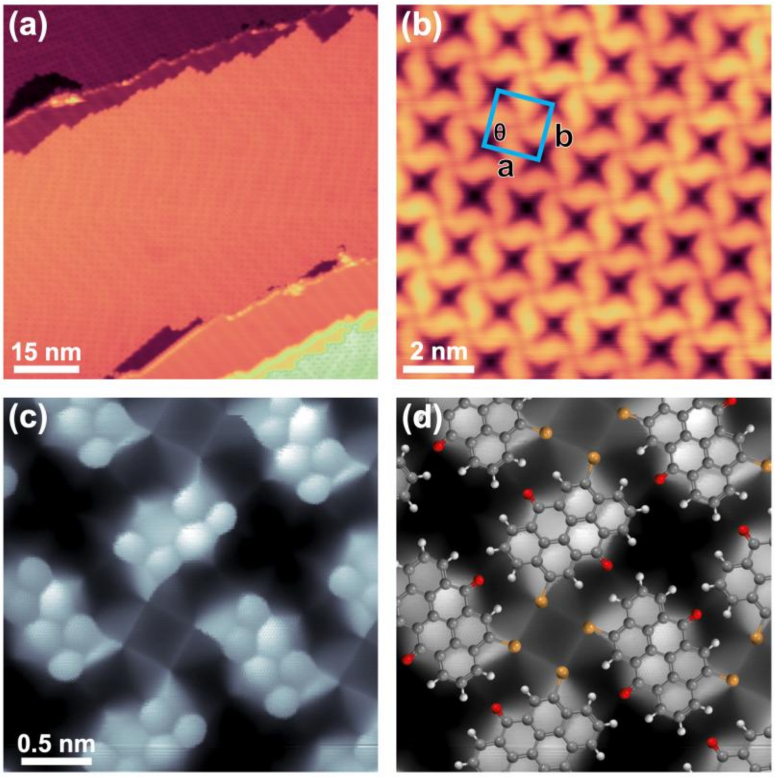

Figure S10. (a) Large-scale STM image of VO3 self-assembled on Au(111) after a deposition at room temperature. The molecular coverage on the large terrace at the centre of the image is close to a full monolayer, with a region of bare Au(111) visible in the upper part of the image. (b) Zoom-in STM image of the assembly pattern. The chirally-segregated islands are found to consist of molecules packed into an approximately square unit cell (unit cell parameters a = b = 1.53 ± 0.04 nm, θ = 89 ± 1°). The σ-holes of the C-Br groups on each molecule are oriented towards the areas of negative electrostatic potential on the sides of the C-Br groups of adjacent molecules. Panels (c) and (d) show a constant-height BR-STM image with overlaid scaled models of the molecule that demonstrates a close fit of the observed features with the molecular structure. The measured Br---Br distance of 3.9 ± 0.1 Å implies that the halogen bond is relatively similar in length to others of the same type (type II, Br---Br) reported in the literature.[13,14] Imaging parameters: (a) I_T = 130 pA, V_b = +1.09 V; (b) I_T = 200 pA, V_b = +0.92 V; (c) constant height, V_b = +0.038 V.

11. STM images of VO3/Au(111) as a function of annealing

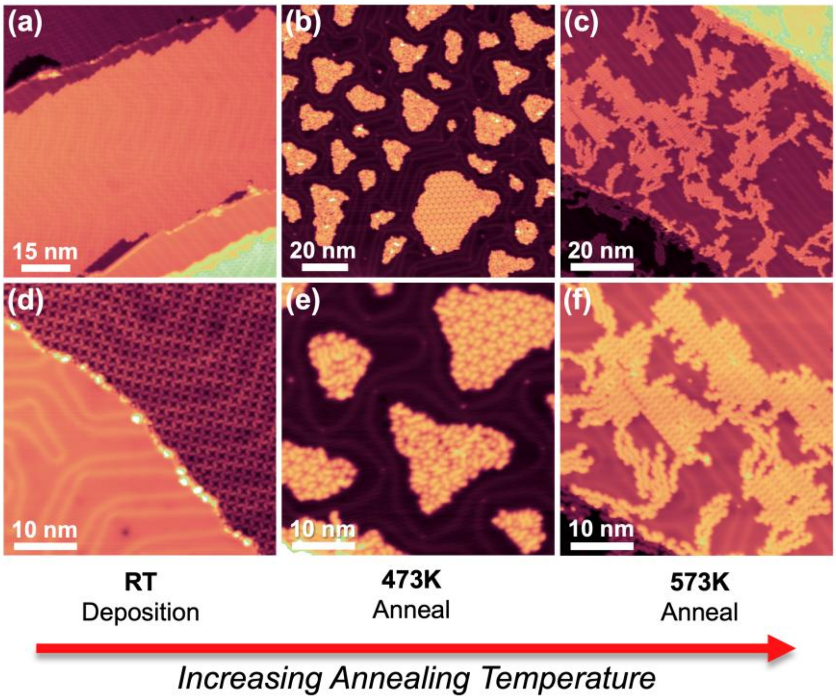

Figure S11. STM images showing the general progression when annealing VO3 on Au(111). After the 473 K anneal, nanoribbons start to form, but they are fairly short and still co-adsorbed with Br atoms. Some regular self-assembled structures are observed, such as the island of dimer nanoribbons/Br in the upper right of panel (e). Annealing temperatures higher than 573 K led to the desorption of most of the Br atoms and to much longer nanoribbons which self-assembled into islands. Imaging parameters: (a) I_T = 130 pA, V_b = +1.09 V; (b) I_T = 150 pA, V_b = +0.92 V; (c) I_T = 270 pA, V_b = +1.09 V; (d) I_T = 200 pA, V_b = +0.92 V; (e) I_T = 320 pA, V_b = +0.65 V; (f) I_T = 270 pA, V_b = +1.09 V.

12. Self-assembly of AO nanoribbons

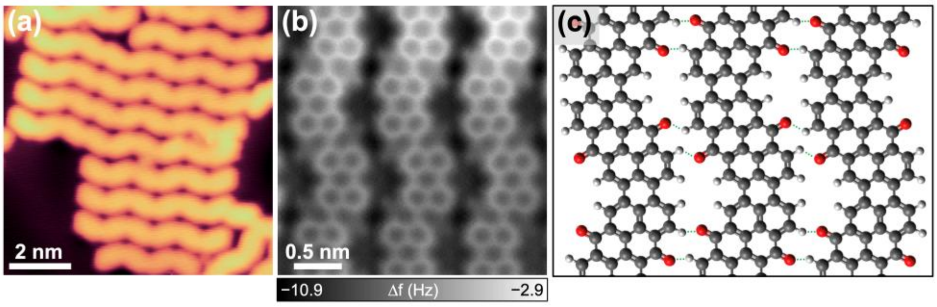

Figure S12. Self-assembly of AO nanoribbons. (a) STM and (b) nc-AFM images of islands of AO nanoribbons formed after annealing VO3 on Au(111) to 573 K. The nanoribbons are no longer co-adsorbed with Br atoms; instead they self-assemble, most likely through weak hydrogen bonds between the ketones and the C-H groups on adjacent nanoribbons. (c) Tentative molecular arrangement of the assembly, with green dashed lines indicating the position of the weak hydrogen bonds. Imaging parameters: (a) I_T = 50 pA, V_b = –0.50 V; (b) oscillation amplitude = 50 pm.

At variance with the PXX nanoribbons, the AO nanoribbons tend to assemble into compact domains following Br desorption. This contrast in behaviour between the two types of ribbons is most probably due to their different intermolecular interactions when adsorbed on Au(111), with the PXX nanoribbons generally seeming to experience a more repulsive interaction with one another. This could be due to a repulsion between stronger interfacial dipoles that are related to a charge transfer with the surface (see Section 16 of the SI), possibly combined with weaker O···H intermolecular interactions.

13. Defective coupling of AO nanoribbons

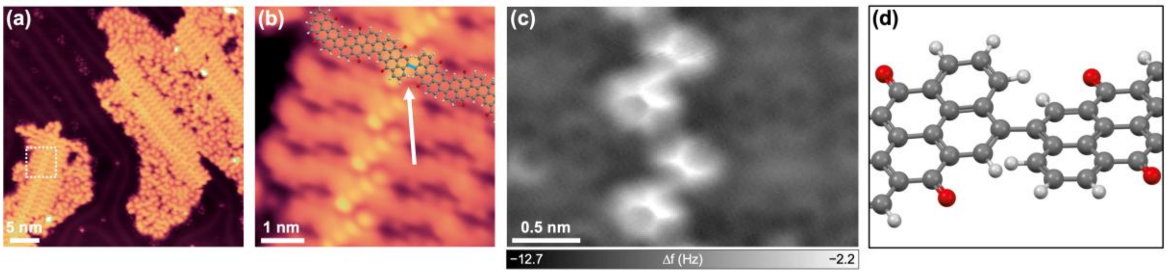

Figure S13. (a) and (b) STM images of AO nanoribbons that display bright junctions connecting their termini with those of neighbouring ribbons. The dashed white box in (a) indicates the position of the zoomed image in (b). These bright features are most probably the signature of single C-C bonds that are formed between two regular nanoribbon sections, as proposed in the molecular model superposed in the upper part of (b). (c) nc-AFM image of another example of the same type of terminal junctions between AO nanoribbons, recorded in a separate experiment. The bright features most probably correspond to rings that are raised out of the plane of the surface. We speculate that these single C-C bonds form instead of the regular fusion between nanoribbon precursors because of their initial misalignment. A regular fusion would require the entire ribbon to flip over in order to achieve the correct orientation, which is clearly energetically too costly. The alternatives to a single C-C bond could be either the formation of two 5-member rings (but this would lead to a very distorted, curved connection), or a single 5-membered ring (which would however lead to a change in the relative orientation of the two nanoribbons, as seen with the kinked PXX nanoribbons). As a consequence, single C-C bonds appear to be the most plausible explanation, with the bright features in the STM and nc-AFM images likely corresponding to the termini of the regular nanoribbon section slightly twisting and rotating around the connecting bond to minimise steric clashes between the C-H groups. This behaviour is depicted in the illustrative model in (d). Imaging parameters: (a) and (b) I_T = 180 pA, V_b = +1.09 V; (c) oscillation amplitude = 50 pm.

14. Positions of dI/dV spectra recorded on short, pure nanoribbons

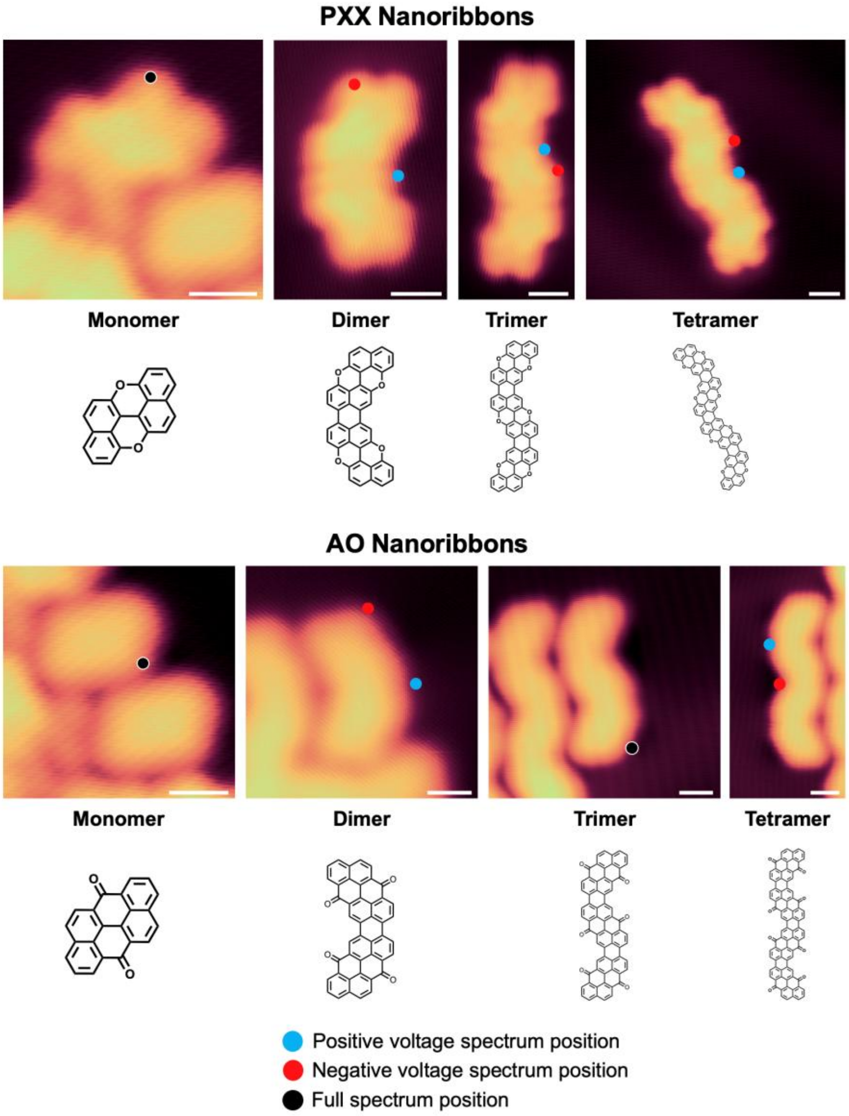

Figure S14. Positions at which the dI/dV spectra were recorded on the monomer, dimer, trimer and tetramer of the PXX and AO nanoribbons shown in Fig. 3. Imaging parameters: all PXX nanoribbons: I_T = 300-350 pA, V_b = –1.00 V. AO nanoribbons: monomer: I_T = 300 pA, V_b = –1.00 V; dimer: I_T = 700 pA, V_b = –1.30 V; trimer: I_T = 100 pA, V_b = –0.50 V; tetramer: I_T = 100 pA, V_b = –0.50 V. Scale bars = 500 pm.

For most of the nanoribbons, the positive and negative bias voltage regions of the spectra were recorded at different positions to maximise the signal from the frontier states. In fact, the occupied and unoccupied states typically exhibit stronger signals over different regions of the molecules due to their spatial localisation, as illustrated in dI/dV images. In the case of the two monomers and the AO trimer, both polarities were recorded in the same position. No unoccupied peaks were observed in the typical voltage range used for STS (± 2.5 V) for the PXX monomer. Increasing the positive bias voltage beyond this range typically led to the destruction of the tip apex and/or the molecule. It should also be noted that dI/dV spectra were generally recorded at the edges of the molecules/nanoribbons, as this is where the signal for the molecular resonances was found to be the strongest.

15. Comparison of dI/dV images recorded with *s*- and *p*-wave tips

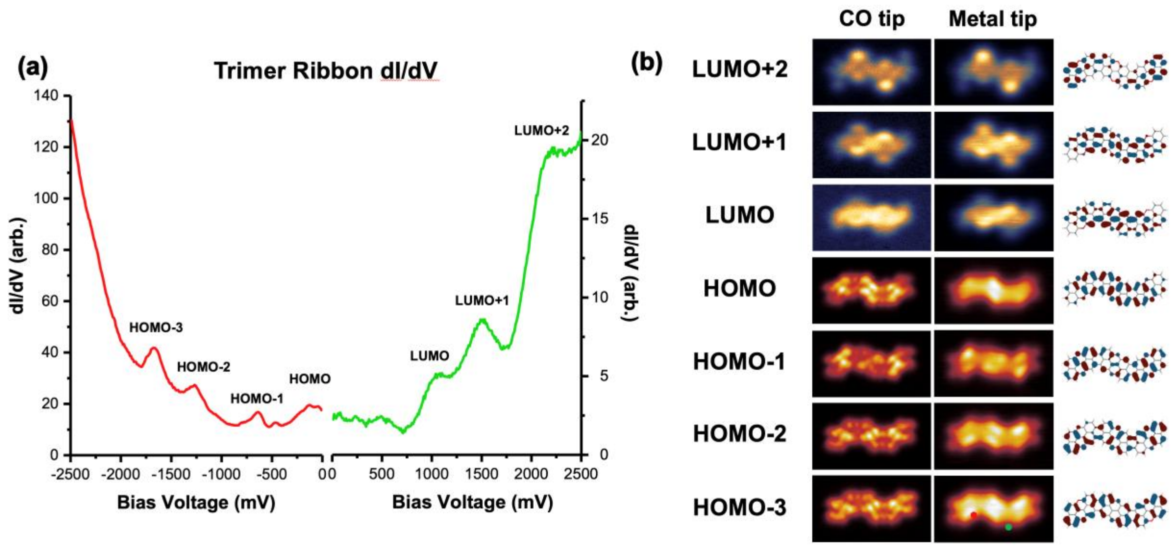

Figure S15. STS measurements on a PXX nanoribbon trimer (different from those shown in Fig. 3(a)). (a) dI/dV spectra recorded at the positions indicated in the lower right panel of (b) by a red and green dot. (b) Constant height dI/dV images recorded at the voltage of each of the observed peaks and measured with both a CO tip (left column) and a metal tip (right column). This allows a comparison between a typical *s*-wave metallic tip and the *p*-wave CO tip. The parts of the molecule with the strongest signal are generally similar for both tips, with finer detail resolved by the CO tip. Density functional theory (DFT)-calculated molecular orbitals are shown alongside the dI/dV images.

16. Energy shift & emptying of the highest occupied state for longer PXX nanoribbons

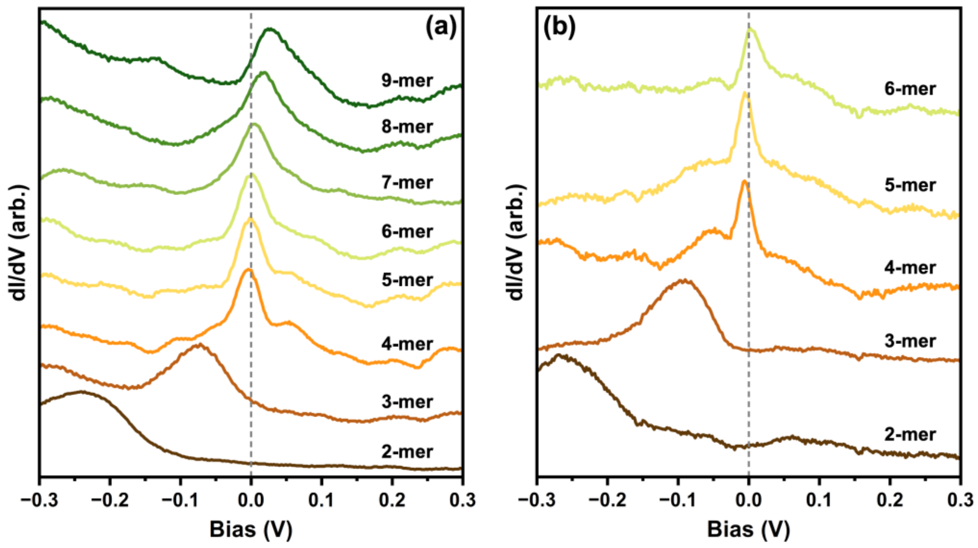

Figure S16. Two sets of dI/dV spectra recorded close to the Fermi level (each set with a different tip) on PXX nanoribbons of different lengths. The set in (b) was also recorded with a smaller bias voltage oscillation (5 mV, compared to 20 mV in (a)) to ensure that the peaks were not broadened by the measurement. The highest occupied state can be seen shifting to higher energies as the nanoribbons increase in length, with the resonance crossing 0 V for nanoribbons around 5 or 6 units long. Above this, the resonance empties and becomes the lowest unoccupied state, with a (presumed) positive charge on the PXX nanoribbons.

The shift of the highest occupied molecular state towards and beyond the Fermi level (Fig. S16), explains the modulation of the contrast observed when attempting to record BR-STM images of longer PXX nanoribbons (e.g. Fig. S7). The tunnelling current signal acquired in BR-STM constant height images derives from a combination of the local density of states and a sharp sub-molecular contrast resulting from the lateral bending of the CO tip due to its repulsive interaction with the atoms and bonds of the molecule.[15] As a consequence, BR-STM images are typically recorded at low bias voltages in order to probe the molecule within its energy gap, without significant influences of the frontier molecular states and with the contrast being largely caused by the internal molecular structure. However, as the PXX nanoribbons become longer, the highest occupied state shifts towards 0 V and the measured features are increasingly affected by the spatial distribution of this state. This is less of an issue for the shorter ribbons (trimers and below) as there are no strong molecular states close to the Fermi level (Fig. S7). In this case, nc-AFM is much better suited to imaging PXX nanoribbons, as the contrast is not derived from the electronic density of states.

It should also be noted that as the highest occupied resonance approaches and passes through 0 V, there may be other effects that contribute to its sharp appearance in the dI/dV spectra. In particular, there may be a transition regime in which the resonance becomes Kondo-like due to partial charge transfer to the surface. However, for longer ribbons the resonance clearly shifts beyond this regime, reaching approximately +40 mV for the nanoribbon shown in Fig. S17. Whilst this transition regime could be interesting to examine in more detail, it lies beyond the scope of the present study and does not affect its general findings.

For the AO nanoribbons, there are no such issues, as both frontier resonances are far enough away from the Fermi level to avoid charge transfer or significant contributions to the current in BR-STM imaging.

17. STS of a 21-unit long PXX nanoribbon

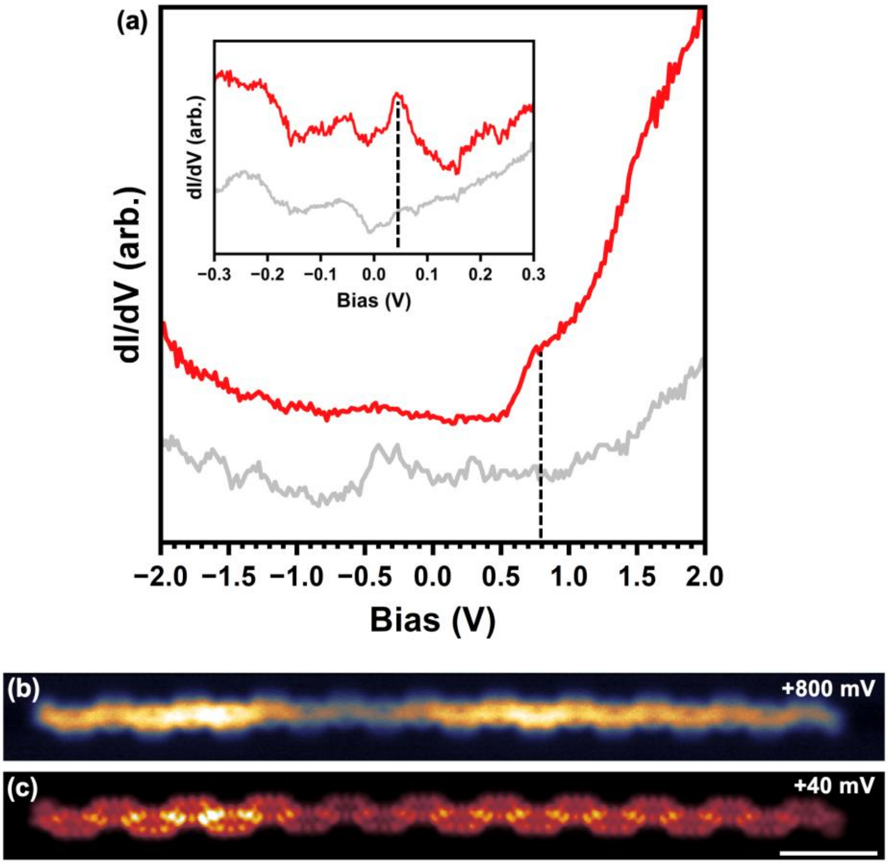

Figure S17. (a) dI/dV spectra and (b, c) constant height dI/dV images (CO tip) of a 21-mer PXX nanoribbon. Reference spectra on the Au(111) surface are shown in light grey. The energy gap in the 'long ribbon limit' is found to be approximately 0.74-0.76 eV, albeit with the highest occupied state having emptied after crossing the Fermi level, with a position of approximately +40 mV. The modulation in the brightness of the images in (b) and (c) is related to the underlying Au(111) herringbone reconstruction, as the nanoribbon was lying across it. Scale bar = 2 nm.

18. Statistical analysis of D/A combinations in mixed dimers

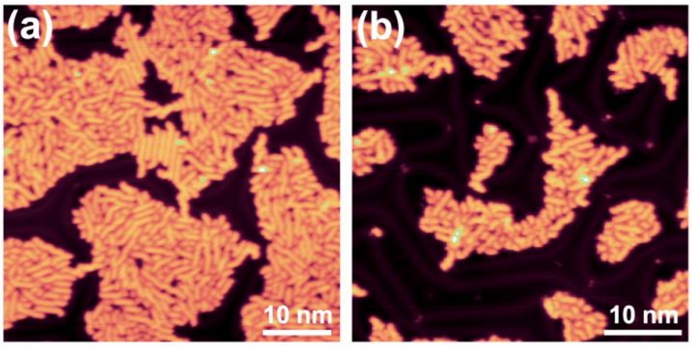

Figure S18. STM images of mixed PXX/AO nanoribbons at (a) high and (b) low molecular coverage, respectively. Imaging parameters: (a) I_T = 190 pA, V_b = +0.53 V; (b) I_T = 140 pA, V_b = +0.53 V.

A number of junctions were examined via BR-STM imaging for the mixed Br_2PXX and VO3 deposition and annealing experiments. The results of the statistical analysis are reported in Table S1. For each sample, the table lists the percentage of each type of junction/connection observed. The number of individual units remaining on the surface but not incorporated into nanoribbons is also reported, followed by the total percentages of each type of constituent unit (PXX or AO) observed for that sample, including both ribbon-incorporated and isolated units.

This analysis reveals that the monomers show a greater tendency to form D-A connections rather than D-D or A-A connections. This effect is particularly pronounced for AO, which is present in excess in both low- and high-coverage experiments.

Table S1. Statistical distribution of junctions within mixed PXX-AO nanoribbons.

**High Coverage Experiment**<br>418 junctions examined		**Low Coverage Experiment**<br>97 junctions examined	
PXX-PXX	25%	PXX-PXX	13%
AO-AO	13%	AO-AO	24%
PXX-AO	62%	PXX-AO	63%
**Total constituent units observed (nanoribbons + isolated)**			
798 monomer units<br>of these: 25 PXX and 166 AO isolated units (not in nanoribbons)		217 monomer units<br>of these: 9 PXX and 56 AO isolated units (not in nanoribbons)	
PXX	43%	PXX	33%
AO	57%	AO	67%

19. Coupling geometries of DD, AA and DA junctions

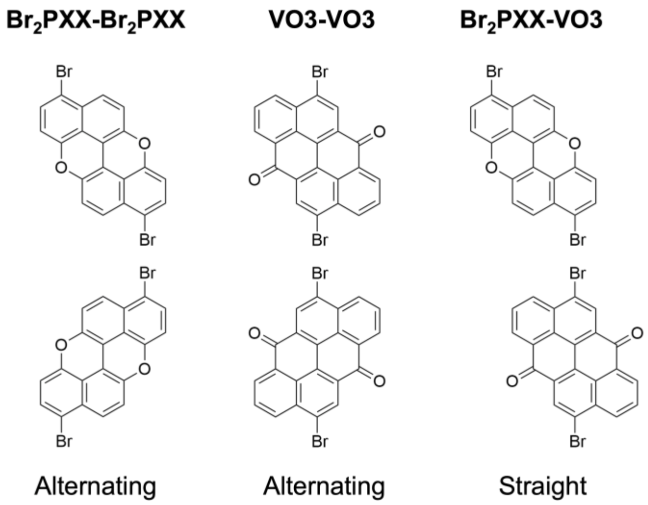

Figure S19. As already noted for the formation of DD and AA junctions in pure PXX and AO nanoribbons, respectively, the relative position of the bromine atoms in the two adjacent precursors that will go on to form the junction is important, as most of the radicals formed during the debromination probably do not ‘hop’ to the other terminal position before reacting. In particular, the best initial configuration seems to be that where the two C-Br (and the two C-H) groups face each other; this may determine the arrangement in an intermediate organometallic step, followed by covalent bond formation. As a consequence, while nanoribbons tend to be characterised by ‘alternating’ junctions when consisting of only PXX or AO (see Fig. S4), most junctions will be ‘straight’ in mixed PXX-AO couplings. This is because the bromines are on different terminal positions for Br_2PXX and VO3.

20. Positions of dI/dV spectra recorded on mixed D-A nanoribbons

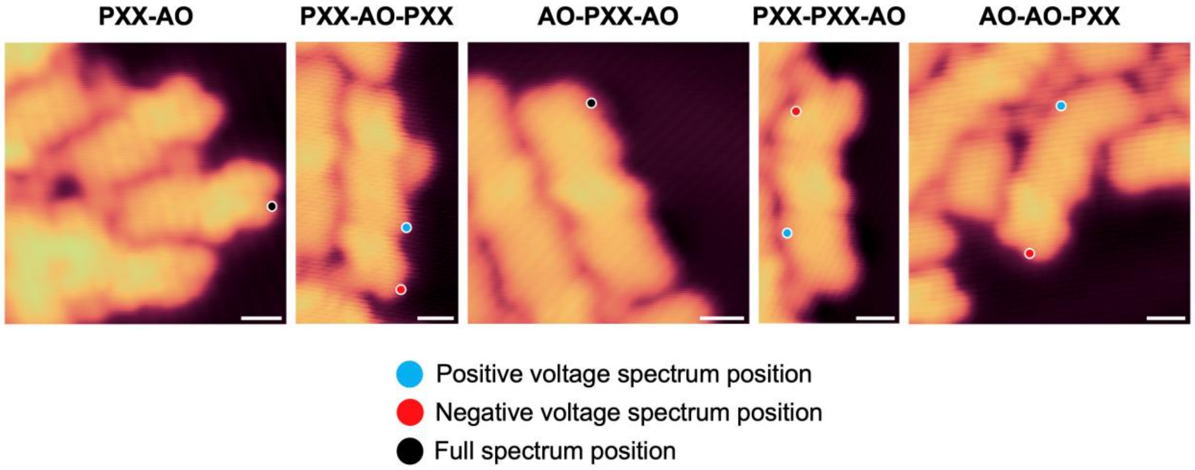

Figure S20. Positions of the dI/dV spectra recorded on the mixed D-A nanoribbons presented in Fig. 4 of the main text. For three of the ribbons, the positive and negative voltage regions of the spectra were recorded in different locations, due to the spatial variations in the intensity of the signals from the occupied and unoccupied states. For example, the highest occupied resonance of the PXX-PXX-AO trimer was more localised to the PXX-PXX section of the trimer, whereas the lowest unoccupied resonance was more localised to the AO section of the trimer. Scale bars correspond to 0.5 nm. Imaging parameters: I_T = 300-320 pA, V_b = –1.00 V.

21. Simple LCMO derivation of HOMO/LUMO energies of mixed D-A trimers

In this section, we introduce an elementary but effective method for estimating the energies of the frontier molecular orbitals of D-A oligomers. This method relies on a highly simplified linear combination of molecular orbitals (LCMO) approach. We start from the energies of the highest occupied molecular orbital (HOMO) and lowest unoccupied molecular orbital (LUMO) of donor (D) and acceptor (A) monomers, respectively, and utilise these values in an iterative *Aufbau* to determine the HOMO and LUMO energies of oligomers composed of these monomers. Specifically, we consider individual PXX (donor) and AO (acceptor) molecules isolated in vacuum, for which DFT calculations yield HOMO energy levels of –4.69 eV and –6.04 eV, respectively and LUMO energy levels of –2.11 eV and –3.78 eV, respectively (see Table S2).

The first step is determining the energies of the frontier molecular orbitals of the three possible dimers. These can be obtained in a variational approximation by using the D and A orbitals as basis set.[16] In particular, for the homodimers DD and AA, the HOMO and LUMO energy levels are given by

$$E_{\mathrm{HOMO_{DD}}} = E_{\mathrm{HOMO_D}} - \beta_{\mathrm{D}} \tag{1}$$

$$E_{\mathrm{LUMO_{DD}}} = E_{\mathrm{LUMO_D}} + \beta_{\mathrm{D}} \tag{2}$$

$$E_{\mathrm{HOMO_{AA}}} = E_{\mathrm{HOMO_A}} - \beta_{\mathrm{A}} \tag{3}$$

$$E_{\mathrm{LUMO_{AA}}} = E_{\mathrm{LUMO_A}} + \beta_{\mathrm{A}} \tag{4}$$

where the β values are the so-called resonance integrals (which have negative values) and where the simplifying assumption has been made that the overlap integrals are negligible.[16]

On the other hand, for the heterodimer, DA, the HOMO and LUMO energy levels are expressed as

$$E_{\mathrm{HOMO_{DA}}} = E_{\mathrm{HOMO_D}} + \frac{(\beta_{\mathrm{DA}})^2}{|\Delta E_{\mathrm{HOMO}}|} \tag{5}$$

$$E_{\mathrm{LUMO_{DA}}} = E_{\mathrm{LUMO_A}} - \frac{(\beta_{\mathrm{DA}})^2}{|\Delta E_{\mathrm{LUMO}}|} \tag{6}$$

where $\Delta E_{\mathrm{HOMO}} = E_{\mathrm{HOMO_D}} - E_{\mathrm{HOMO_A}}$; $\Delta E_{\mathrm{LUMO}} = E_{\mathrm{LUMO_D}} - E_{\mathrm{LUMO_A}}$; β_{DA} is the corresponding resonance integral; also in this case, the overlap integral is assumed to be zero.[16]

Calculating the resonance integrals β requires knowledge of the molecular orbital wave functions for the D and A monomers and of the expression of the system Hamiltonian. However, since here we are only interested in the overall qualitative trends of the frontier orbital energies of D-A oligomers as a function of their monomer sequence, we adopt a simple assumption for β. Specifically, β is taken as 0.2 eV for larger ΔE_{HOMO} or ΔE_{LUMO} energy differences (> 1 eV) and 0.08 eV for smaller energy differences (< 1 eV). A constant value of 0.2 eV is also used for all homodimers (Equations (1)-(4)). *A posteriori*, this appears to be a reasonable choice of β values when comparing the approximated LCMO energy values with those obtained by DFT. With this approximation, Equations (1-6) can be simply calculated resulting in the values reported in Table S2.

Table S2. HOMO and LUMO energy levels of short D-A oligomers, as obtained from the STS measurements, DFT calculations (HSE06) and the highly simplified LCMO approach described in this Section. All values are in eV.

	**STS**			**DFT (HSE06)**			**simplified LCMO**			
oligomer	HOMO	LUMO	gap	HOMO	LUMO	gap	composition	HOMO	LUMO	gap
D	–0.76	–	–	–4.69	–2.12	2.57	D	–4.69	–2.12	2.57
A	–1.91	1.11	3.02	–6.04	–3.78	2.26	A	–6.04	–3.78	2.26
DD	–0.24	1.40	1.64	–4.3	–2.82	1.48	(D)(D)	–4.49	–2.32	2.17
AA	–1.26	0.78	2.04	–5.67	–4.28	1.39	(A)(A)	–5.84	–3.98	1.86
DA	–0.70	0.96	1.66	–4.92	–3.71	1.21	(D)(A)	–4.66	–3.80	0.86
DDD	–0.07	1.10	1.17	–4.19	–3.02	1.17	(DD)(D)	–4.46	–2.35	2.11
DAD	–0.53	0.83	1.36	–4.67	–3.63	1.04	(D)(AD)	–4.46	–3.81	0.66
DDA	–0.26	0.90	1.16	–4.57	–3.69	0.88	(DD)(A)	–4.44	–3.83	0.62
ADA	–0.60	0.86	1.46	–5.06	–4.06	1.00	(AA)(D)	–4.66	–4.00	0.65
AAD	–0.64	0.68	1.32	–4.99	–4.12	0.87	(A)(DA)	–4.63	–4.07	0.56
AAA	–1.18	0.57	1.75	–5.59	–4.43	1.16	(A)(AA)	–5.81	–4.01	1.80

Aside from the precise numeric values, it is particularly instructive to understand how the functional dependences expressed in Equations (1-6) determine the HOMO and LUMO energies of the dimers relative to the relative energetic positions of the HOMO and LUMO of the constituent monomers. In the case of the two homodimers, AA and DD, respectively, what can be seen in Fig. S22(a) and Fig. S22(f) is that the HOMO-LUMO gap is reduced almost symmetrically with the respect to that of the monomers. However, the situation is rather different in the case of the DA heterodimer. Here Fig. S21(a) shows that the hybridisation of the frontier molecular orbitals of the D and A moieties results in the LUMO of the DA dimer being energetically close to the LUMO of A (and mostly independent of the energetic position of the LUMO of D), while the energy of the HOMO of the DA dimer is close to that of the HOMO of D (and mostly independent of the energy of the acceptor HOMO). This is because of the large values of ΔE_{HOMO} and ΔE_{LUMO} in Equations (5) and (6), resulting in rather small corrections to $E_{\mathrm{HOMO}_\mathrm{D}}$ and $E_{\mathrm{LUMO}_\mathrm{A}}$, respectively. As mentioned in the main paper, this achieves two important goals in the design of optoelectronic molecular materials: i. the ability of fine tuning of the energy levels because the HOMO and LUMO of the resulting DA dimer can be modified independently of each other, and ii. the narrowing of the bandgap.

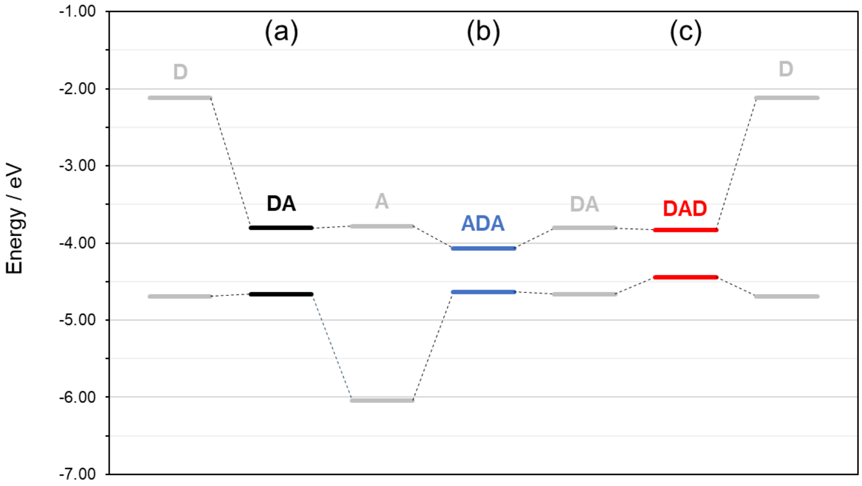

Figure S21. Energy level diagram showing the energetic position of the HOMO and LUMO of the A and D monomers, as well as those of (a) the DA, (b) the ADA and (c) the DAD oligomers, obtained through the iterative *Aufbau* based on the elementary LCMO approach discussed in the text.

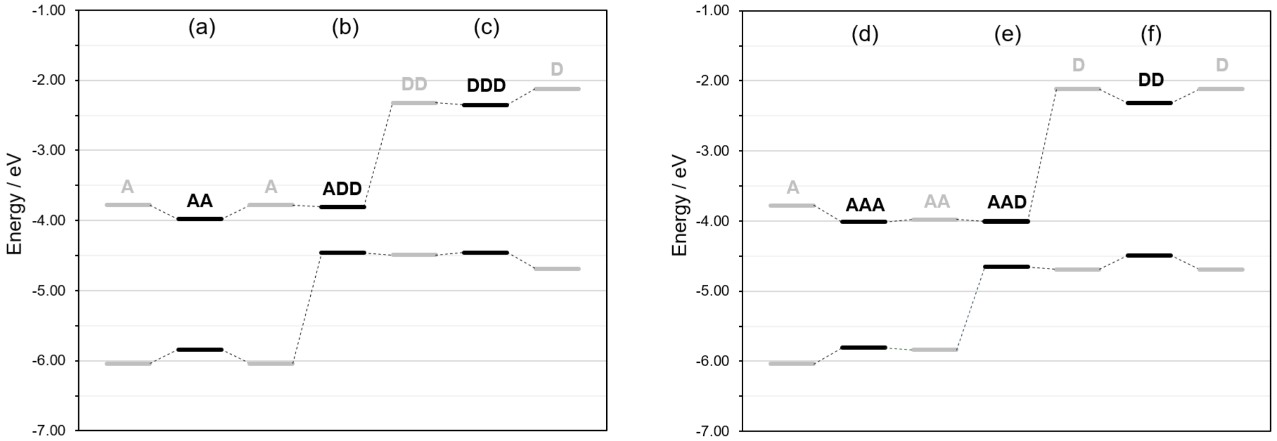

Figure S22. Energy level diagrams showing the results of the elementary LCMO approach discussed in the text, when applied to calculating the energetic position of the HOMO and LUMO levels of the two homodimers and the two homotrimers (AA, DD, AAA and DDD), as well as for a different *Aufbau* of the two mixed trimers, ADD and AAD, respectively.

The energies of the HOMO and LUMO levels for the six possible distinct trimers can be calculated by adopting a similar variational approach and combining the HOMO and LUMO energies levels of dimers and monomers. In this case, since the HOMO and LUMO energies of the dimers and monomers are different, only Equations (5) and (6) for the non-degenerate case are needed (or, more precisely, the equivalent equations for the corresponding combinations of monomers and dimers). The results of these calculations are also reported in Table S2. However, also in this case, the more instructive aspect of this exercise is understanding how the final HOMO and LUMO energies depend on the relative positions of the HOMO and LUMO levels of the constituent monomers and dimers. So, for example, the DAD trimer can be obtained by combining a DA dimer with a D monomer (see Fig. S21(c)). Let us start by analysing the HOMO level, resulting from the combination of $E_{\mathrm{HOMO_{DA}}}$ and $E_{\mathrm{HOMO_{D}}}$. These two values are relatively close to each other and thus their hybridisation results in a significantly upshifted HOMO level for the DAD trimer, because of the small value of ΔE_{HOMO} at the denominator in Equation (5). On the contrary, since the $E_{\mathrm{LUMO_{DA}}}$ and $E_{\mathrm{LUMO_{D}}}$ values are quite different, the resulting hybridised $E_{\mathrm{LUMO_{DAD}}}$ value is quite close to $E_{\mathrm{LUMO_{DA}}}$, the smallest of the two (see Fig. S21(c)). The situation is mirrored for the ADA trimer obtained by combining a DA dimer with an A monomer. Here, $E_{\mathrm{HOMO_{ADA}}}$ is quite close to $E_{\mathrm{HOMO_{DA}}}$, while $E_{\mathrm{LUMO_{ADA}}}$ is significantly lower than the two quite similar $E_{\mathrm{LUMO_{DA}}}$ and $E_{\mathrm{LUMO_{A}}}$ values (see Fig. S21(b)). Finally, the homotrimers have their HOMO (LUMO) levels higher (lower) than the corresponding homodimers (see Fig. 22(c) and Fig. 22(d) for DDD and AAA, respectively), although the bandgap reduction is smaller than when moving from monomer to dimer, similarly to what predicted by DFT (see Fig. S2(b)).

Both the DFT results and the experimental measurements indicate that the energy levels of the trimers are predominantly determined by the quantity of distinct D and A monomers they comprise. However, the arrangement of these monomers also has a secondary impact on the energetic positions of HOMO and LUMO levels (see Fig. 4 in the main paper). On the contrary, in the simple LCMO discussed here, the frontier energies levels of a trimer only depend on those of the dimer and the monomer it is composed of, not on the order these are combined together. So, for example, the energy levels of (A)(AD) are predicted to be identical to those of (A)(DA). This presents a limitation of this approach, albeit not a significant one given that, as previously noted, our focus lies solely on the general

qualitative patterns. Another problem is that the energy levels of a trimer might depend on what monomer and dimer are used for its composition. So, for example, a DDA trimer can be obtained, as we have seen above, by combining a D monomer with a DA dimer as (D)(DA) (Fig. S21(c)). However, the same trimer could also result from joining a DD dimer with an A monomer as (DD)(A) (Fig. S22(b)). As is shown in Table S2, these two different paths result in slightly different values for the HOMO and LUMO energies. However, this is effectively irrelevant when looking only for qualitative trends, since the energetic differences are extremely small (within a few percent).

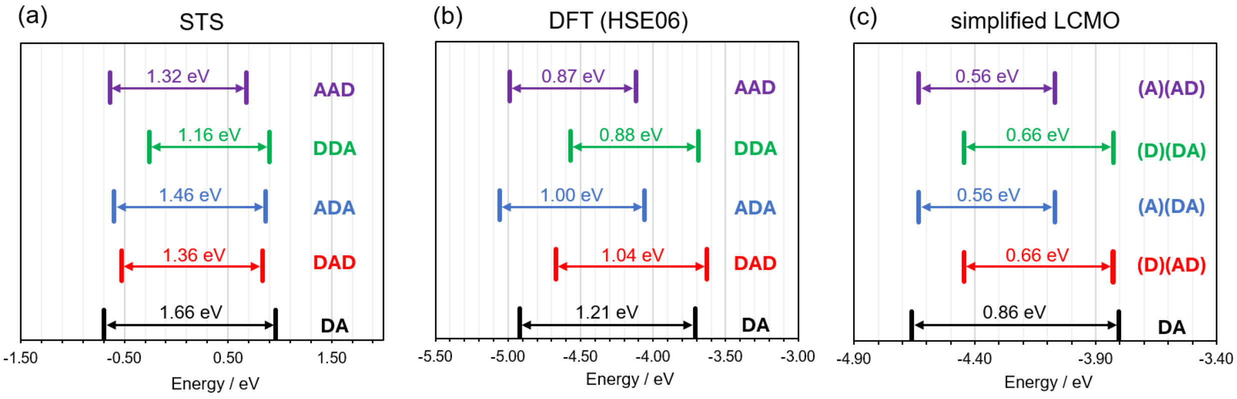

Figure S23. Direct comparison between the energetic positions of the HOMO and LUMO levels and the energy gaps as obtained from (a) the STS experiments, (b) DFT calculations and (c) simple LCMO model described in this Section.

Figure S23 presents a comparative analysis of the energetic positions of HOMO and LUMO levels, along with the energy gaps derived from STS experiments, DFT calculations and the simplified LCMO model described above. This direct graphic comparison clearly highlights that, despite its highly simplified nature, the proposed LCMO model qualitatively captures all essential features and trends of the energy of the frontier molecular orbitals of D-A dimers and trimers. These trends remain consistent regardless of the specific choice of resonance integrals β, within a broad range of plausible values. Furthermore, the model provides an intuitive framework for interpreting and, importantly, predicting these trends. By applying an analogous approach to that used for trimers, this simplified model helps elucidate and rationalise the design principles required to achieve a desired electronic structure in an arbitrary D-A oligomer, without the need of complex or computationally demanding methods.

Finally, it should be noted that the apparently large discrepancy (4–5 eV) between the experimental and calculated absolute HOMO and LUMO energies reported in Table S2 arises from the fact that the two datasets are referenced to different energy zeroes. The STS values are measured relative to the Fermi level of the Au(111) substrate, whereas the DFT (HSE06) and LCMO results (directly derived from the DFT calculations) correspond to isolated oligomers in the gas phase and are therefore naturally referenced to the vacuum level. As a consequence, the absolute values differ approximately by the work function of Au(111), which is about 5.2–5.3 eV, in addition to any interface-induced renormalisation effects.

As an illustrative example, the AO monomer (A) exhibits STS resonances at −1.91 V (HOMO) and +1.11 V (LUMO) relative to the Fermi level, while the corresponding gas-phase HSE06 values are −6.04 eV and −3.78 eV relative to vacuum. After accounting for the Au(111) work function, the experimental levels correspond to approximately −7.2 eV and −4.2 eV on the vacuum scale, indicating stabilisation of about 1.2 eV and 0.4 eV, respectively, compared to the gas-phase HSE06 energies.

Comparable shifts are obtained for the other oligomers listed in Table S2. This degree of stabilisation is entirely consistent with expectations for molecules adsorbed on a polarisable metal substrate. Image-charge screening, interface dipoles and possible charge transfer are well known to lower the energies of molecular cationic and anionic states and to reduce frontier energy gaps by approximately 1–2 eV for conjugated molecules on metal or graphite surfaces.

22. Variability of dI/dV spectra recorded on PXX-AO nanoribbon dimers

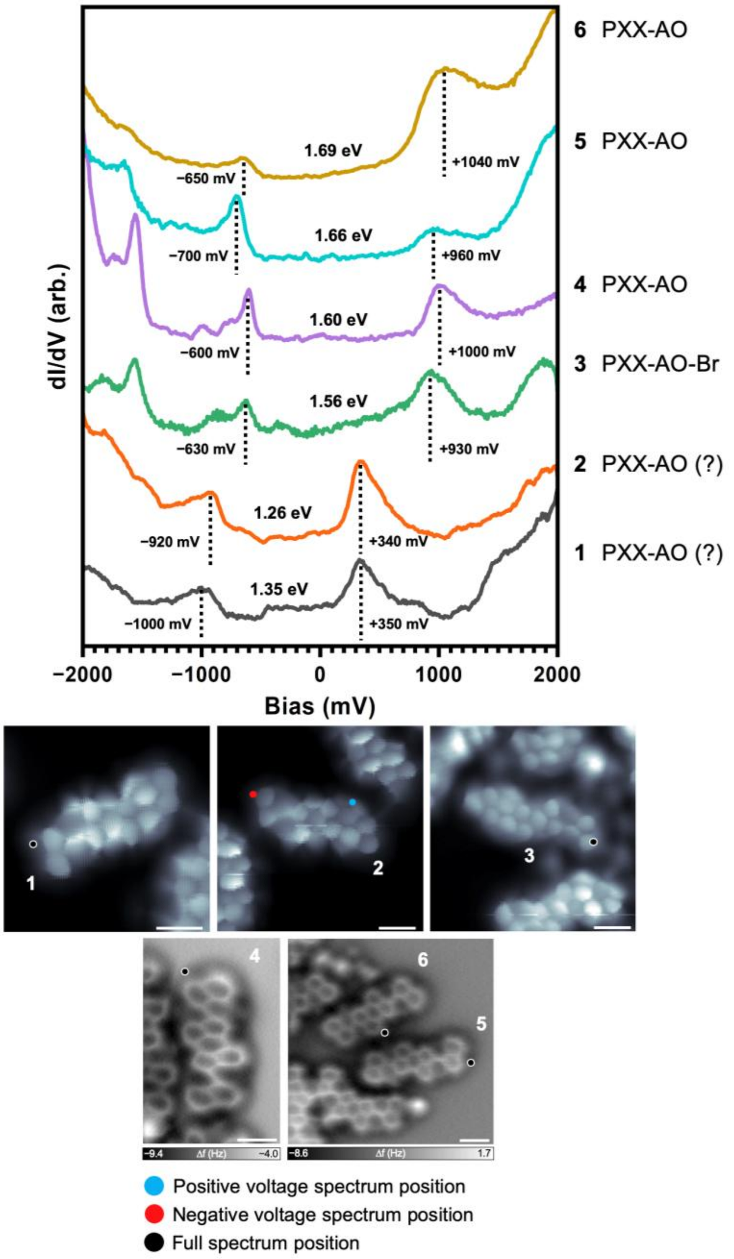

Figure S24. dI/dV spectra recorded for 6 different PXX-AO dimers with varying terminations and surroundings. The locations at which these spectra were acquired are indicated in the constant height BR-STM and nc-AFM images in the lower panels. The approximate positions of their frontier resonances, along with the resulting energy gaps, are indicated in the spectra. All BR-STM images recorded at V_b = +0.04 V. nc-AFM images recorded with an oscillation amplitude of 50 pm.

Two distinct types of mixed dimer can be identified from the dI/dV data shown above. Dimers 1 and 2 exhibit downshifted energy levels and a narrower energy gap. In contrast, dimers 3-6 display their highest occupied resonance between –600 and –700 mV and their lowest unoccupied resonance at around +1000 mV. Dimer 3 had a Br attached at one end, which may account for slight differences in the positions of its frontier resonances. We believe that dimers 3-6 are more reliable indicators for the positions of the frontier resonances and the energy gap, which is why one of these is included in Fig. 4 of the main text. This is because their energy gaps and HOMO/LUMO positions fit much more closely to those expected from the DFT calculations. Three of these dimers were also imaged with nc-AFM, helping to confirm that they did not have any terminations or differences in their structure/bonding (e.g. an oxygen or carbon bound to a metal atom) that could have caused energy shifts. It may be something akin to this that caused the shifts for dimers 1 and 2, but this could not be confirmed *via* nc-AFM imaging at the time. Both of these dimers had a ketone group pointing towards the ketone moiety of an adjacent nanoribbon, potentially forming a metal-organic structure that could affect their electronic properties, as has been observed for ketone-functionalised chiral graphene nanoribbons.[2] Generally, the energy gap for dimers 3-6 was measured to be around 1.6 eV. Scale bars = 0.5 nm.

23. Effect of environment on dI/dV resonances of Br_2PXX molecules

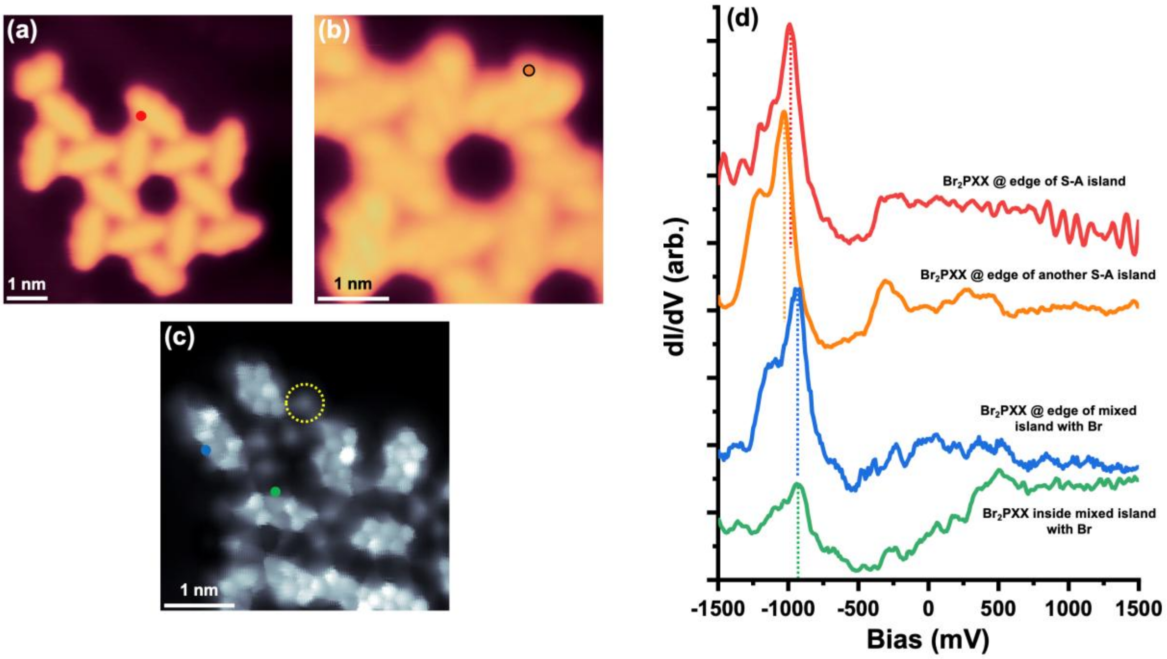

Figure S25. dI/dV spectra recorded on Br_2PXX molecules in various environments, demonstrating the potentials for peak shifts induced by changes in the surroundings, even without altering the molecule's chemical structure. (a) and (b) show the molecules in two similar situations at the edge of self-assembled islands, with slightly different HOMO peak positions (–1030 mV *vs.* –990 mV). When the molecules self-assemble with bromine atoms and other molecules (panel (c)), a stronger up-shift can occur, with both highlighted molecules showing a HOMO peak at –940 mV. Consequently, an up-shift of approximately +100 mV can be reasonably anticipated when bromine atoms are locally adsorbed alongside other molecules in this study, including the mixed nanoribbons. (d) dI/dV spectra recorded at the positions indicated by the coloured dots in the images on the left, with each spectrum colour-matched to its corresponding position. Imaging parameters: (a) I_T = 190 pA, V_b = +1.06 V; (b) I_T = 190 pA, V_b = –1.06 V; (c) constant height, V_b = +0.03 V.

24. A comparison between images recorded with two different CO tips

A variation in the appearance of dI/dV images of the same molecular electronic states was sometimes found between different CO tips during experiments. One CO tip that was capable of achieving bond-resolution, albeit with a slight distortion (tip 1 in Fig. S26)), displayed features in dI/dV images that were much more similar to what would be expected for a tip with more *s*-wave character (closer to the calculated molecular orbitals). This is most probably related to the structure of the metal tip 'above' the adsorbed CO molecule. The nc-AFM and dI/dV images in Fig. 4(f) and (g) were recorded with this tip, whilst the rest were recorded with another (tip 2). Tip 2 showed less distortion of the molecular structure in the nc-AFM images and more *p*-wave type features in the dI/dV images. A comparison between the two tips is shown below in Fig. S26. When simulating the dI/dV images shown in Fig. 4, two different ratios for *s*-wave:*p*-wave character were utilised. For (d), (e) and (h), a probe with 25% *s*- and 75% *p*-wave character was used to model "Tip 2". For (f) and (g), a probe with 75% *s*- and 25% *p*-wave character was used instead for "Tip 1".

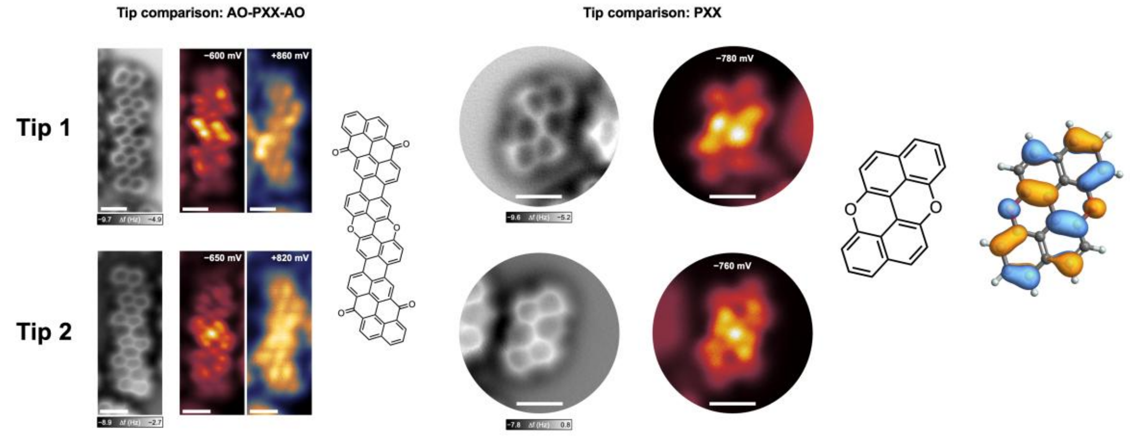

Figure S26. Comparison between two different CO-terminated tips, with nc-AFM and constant-height dI/dV images of an AO-PXX-AO trimer and a PXX monomer. Tip 2 had less distorted imaging in nc-AFM with a much more apparent *p*-wave character in the dI/dV imaging. This was particularly demonstrated by a strong bright signal at the centre of PXX units for the highest occupied resonances of both molecules, corresponding to the position of a central node in their HOMOs (gas-phase DFT calculated HOMO of PXX is shown on the right). Scale bars = 0.5 nm. Nc-AFM images recorded with an oscillation amplitude of 50 pm.

25. Differentiating between the functional groups with bond-resolving techniques

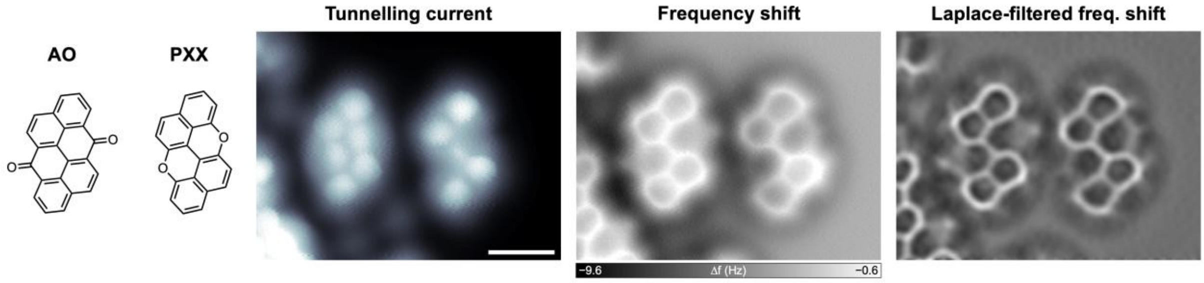

Figure S27. A constant-height image (CO tip) of an AO unit alongside a PXX unit. The tunnelling current signal is shown in (a), with the simultaneously recorded frequency shift signal shown in (b). A Laplace-filtered form of the frequency shift signal is shown in (c) as a way of enhancing the contrast. The original data was filtered with an initial Gaussian smoothening, followed by a Laplace filter, another Gaussian smoothening and a colour inversion. The C=O and C-O-C groups can be clearly differentiated in all three images. The PXX unit also serves as an example of the difference in the observed contrast that occurs when a molecule is adsorbed with another molecule on one side, with this side appearing slightly darker in the nc-AFM image. Scale bar = 0.5 nm.

26. DFT calculations - Influence of the exchange-correlation functional

To assess how the electronic properties of the D–A nanoribbons depend on the choice of the exchange-correlation (XC) functional, we performed a series of DFT calculations using three representative functionals: B3LYP,[17] vdW-DF,[18] and HSE06.[19] Table S3 summarises the resulting HOMO and LUMO energy levels, as well as the corresponding band gaps, for nanoribbons with varying D-A composition.

All XC functionals consistently reproduce the expected trends in frontier orbital alignment, highlighting the sensitivity of the electronic structure to both monomer composition and nanoribbon length. Specifically, increasing the donor content raises the HOMO energy, while increasing the acceptor content lowers the LUMO, with an overall narrowing of the band gap as the nanoribbons become longer. This behaviour reflects the modular tunability inherent in D–A copolymer architectures.

Compared to experimental values (Table S2), B3LYP tends to overestimate the gap, while vdW-DF underestimates it. The HSE06 hybrid functional yields intermediate results, showing the closest agreement with the experimentally measured dI/dV spectra. Consequently, all DFT results discussed in the main text are based on calculations using HSE06.

Table S3. HOMO and LUMO energy levels (in eV) and corresponding band gaps of selected donor–acceptor nanoribbons, calculated using three different exchange-correlation functionals: B3LYP, vdW-DF, and HSE06. In the nanoribbon labels, the letters indicate the monomer type (PXX, AO, AA, or armchair, AR) and the number denotes the number of repeat units.

	B3LYP			VdW-DF			HSE06		
nanoribbon	HOMO	LUMO	Band gap	HOMO	LUMO	Band gap	HOMO	LUMO	Band gap
PXX1	−4.98	−1.67	3.31	−4.54	−2.42	2.12	−4.69	−2.12	2.57
PXX2	−4.53	−2.39	2.15	−4.21	−3.01	1.20	−4.30	−2.82	1.48
PXX3	−4.4	−2.60	1.80	−4.12	−3.18	0.94	−4.19	−3.02	1.17
AO1	−6.3	−3.32	2.98	−5.83	−3.97	1.86	−6.04	−3.78	2.26
AO2	−5.86	−3.83	2.03	−5.53	−4.39	1.13	−5.67	−4.28	1.39
AO3	−5.76	−4.00	1.76	−5.49	−4.52	0.94	−5.59	−4.43	1.16
AA1	−5.16	−2.27	2.89	−4.77	−2.89	1.88	−4.88	−2.64	2.23
AA2	−4.73	−2.83	1.90	−4.45	−3.36	1.09	−4.51	−3.19	1.31
AA3	−4.55	−3.05	1.50	−4.31	−3.52	0.79	−4.35	−3.39	0.96
AA4	−4.46	−3.18	1.28	−4.25	−3.61	0.64	−4.28	−3.50	0.78
AR2	−5.21	−2.18	3.03	−4.79	−2.85	1.94	−4.91	−2.60	2.31
AR4	−4.64	−2.81	1.83	−4.36	−3.36	1.01	−4.41	−3.20	1.22
AR6	−4.40	−3.07	1.33	−4.20	−3.54	0.66	−4.22	−3.42	0.80
AR8	−4.27	−3.22	1.05	−4.12	−3.64	0.47	−4.12	−3.54	0.58

27.Synthetic method

All chemicals and solvents were purchased from Sigma Aldrich, TCI chemicals, Acros Organics and Fluorochem and were used as received. CuO was dried prior to the reaction at 100 °C. ^{1}H- and ^{13}C-NMR spectra were recorded on *Varian Inova* or *400* MHz NMR spectrometer. Chemical shifts (δ) are reported in parts per million and solvent residual peaks are used as internal standard. High resolution matrix assisted laser desorption ionisation was performed using a Waters Synapt G2-Si time of flight mass spectrometer.

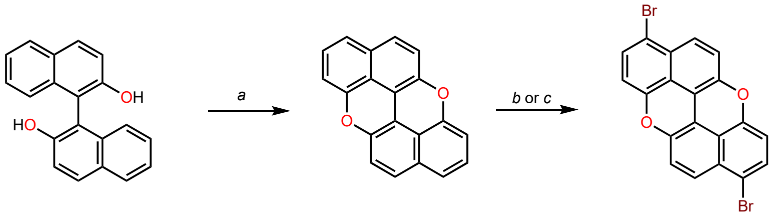

Figure S28. Synthesis of PXX and 3,9-Br_2PXX derivative. Reagents and conditions: (a) CuO, 220 °C, 3 h; (b) Br_2, anhydrous CH_2Cl_2, N_2, −78 °C 1h→ rt 2h; (c) *N*-bromomosuccinimide, *o*-dichlorobenzene, 180 °C, 6 h.

***Peri*-xanthenoxanthene** (**PXX**). This compound was prepared according to a modified literature procedure.[20] A mixture of 1,1'-bi-2-naphthol (2.0 g, 7.0 mmol) and CuO (1.2 g, 15.4 mmol) in nitrobenzene (2.0 mL) was stirred at 220 °C for 3 hours, open to air. Then, the nitrobenzene was distilled off and the residue was filtered through a short silica pad with $CHCl_3$ as eluent. Pure product was obtained as bright yellow needles (1.2 g, 61% yield) after sublimation.

^{1}**H-NMR** (500 MHz, Benzene-d_6): δ 6.88 (d, *J* = 9.0 Hz, 2H), 6.81 (d, *J* = 2.1 Hz, 2H), 6.80 (s, 2H), 6.68 (d, *J* = 9.0 Hz, 2H), 6.56 (dd, *J* = 5.2, 3.3 Hz, 2H). ^{13}**C-NMR** (126 MHz, Benzene-d_6): δ 153.18, 144.70, 131.76, 127.35, 126.55, 122.12, 120.36, 117.49, 112.00, 108.95. **HRMS** (TOF EI+): found $C_{20}H_{10}O_2$ requires 282.0681, found 282.0682.

3,9-Dibromo-*peri*-xanthenoxanthene (**3,9-Br$_2$PXX**). This compound was prepared according to two modified literature procedures.[21,22]

Procedure 1. To a stirred suspension of PXX (0.5 g, 1.8 mmol) in anhydrous CH_2Cl_2 (50 mL), at −78 °C under an N_2, was added a solution of bromine (0.6 g, 3.5 mmol) in anhydrous CH_2Cl_2 (10 mL), over 30 minutes. The solution was stirred for 1 hour and left to warm-up to RT, and stirred for 2 more hours. The reaction mixture was poured into a saturated aqueous solution of $NaHSO_3$, and stirred for 1 hour. The mixture was filtered and washed with H_2O, CH_3CH_2OH, and CH_2Cl_2. Product was obtained as yellow needles after re-crystallization from hot *o*-DCB (0.6, 77% yield).

Procedure 2. A stirred suspension of PXX (0.5 g, 1.8 mmol) and NBS (0.6 g, 3.6 mmol) in *o*-DCB was stirred at 180 °C, for 6 hours. The reaction mixture was left to cool down to RT and was diluted with CH_3CH_2OH. The mixture was filtered, washed with CH_3CH_2OH and dried. Product was obtained as yellow needles after re-crystallization from hot *o*-DCB (0.6, 82% yield).

^{1}**H-NMR** (400 MHz, Benzene-d_6): δ 7.44 (d, *J* = 9.2 Hz, 2H), 7.04 (d, *J* = 8.2 Hz, 2H), 6.64 (d, *J* = 9.2 Hz, 2H), 6.20 (d, *J* = 8.2 Hz, 2H). ^{13}**C-NMR** was not recorded due to the low solubility of this compound. **HRMS** (TOF EI+): found $C_{20}H_8Br_2O_2$ requires 439.8871, found 439.8768. *Note*: by-products, which include the dibromo regioisomer or the tribrominated molecule were observed in STM experiments even after extensive purification procedures – including Soxhlet extraction with THF or benzene and even after 10 re-crystallization cycles using slow cooling from boiling *o*-DCB.

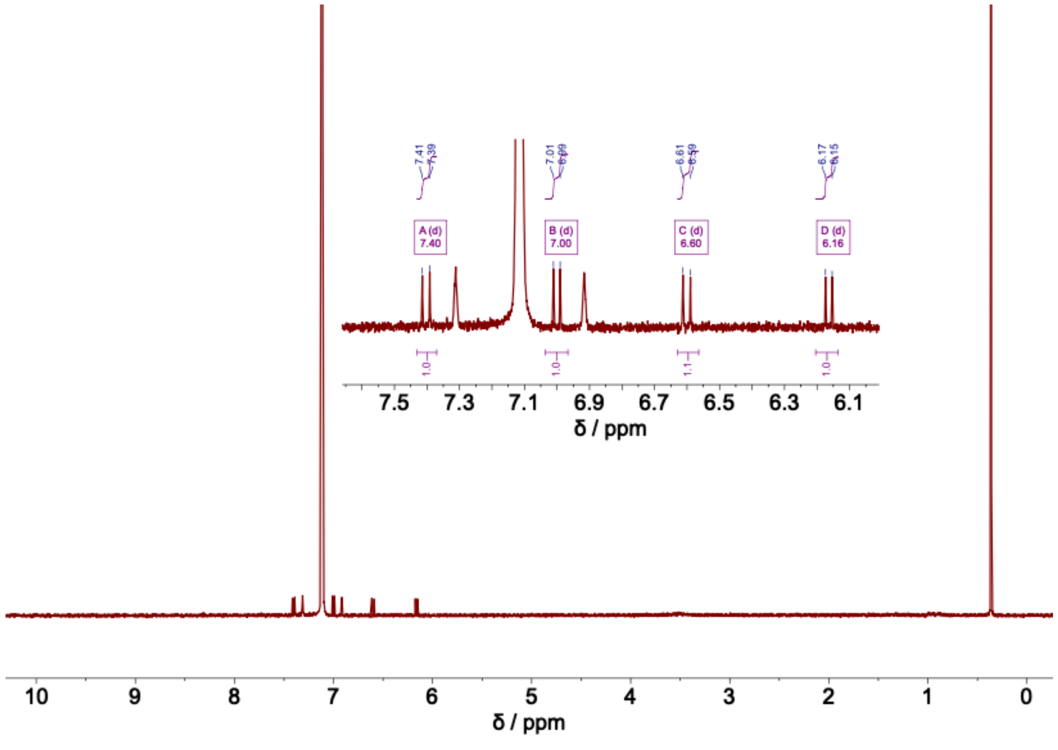

Figure S29. ^{1}H-NMR (Benzene-d_6, 298 K) of 3,9-Br$_2$PXX.

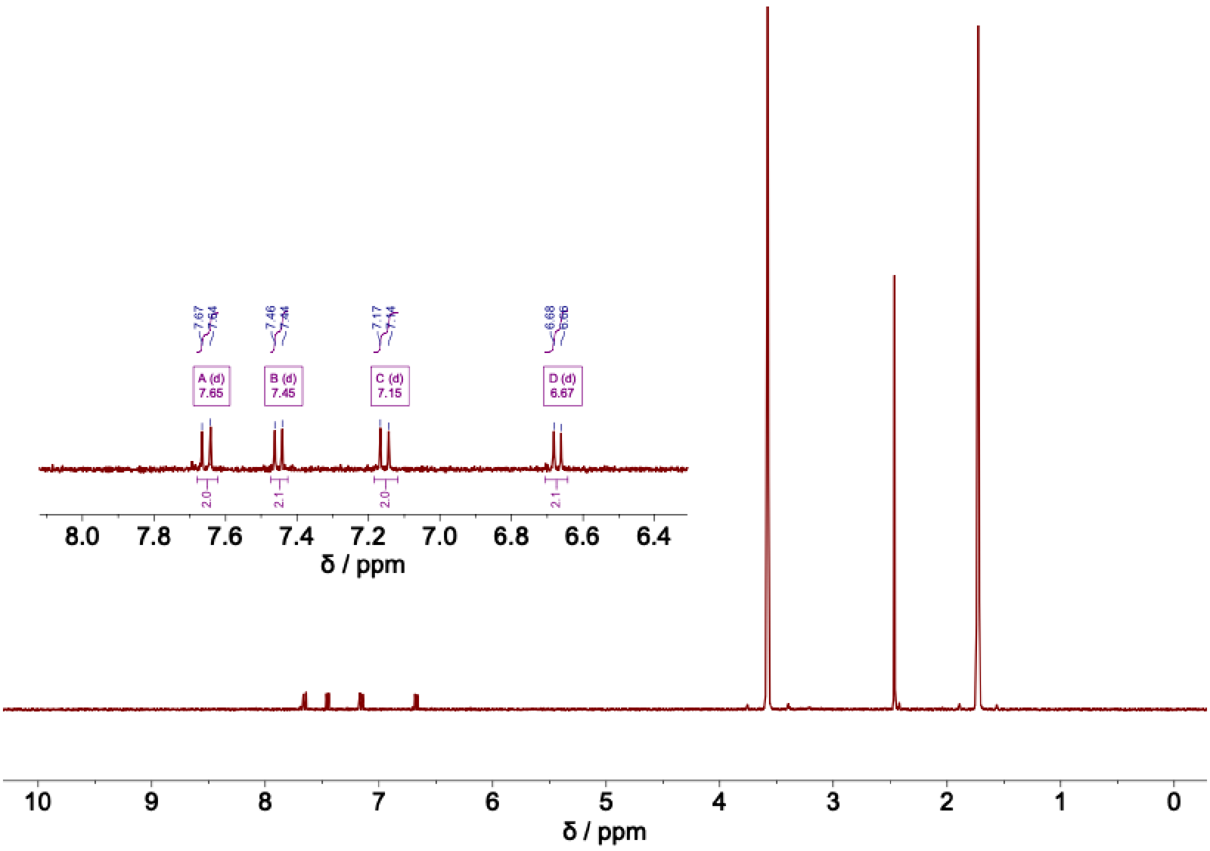

Figure S30. ^{1}H-NMR (Tetratdrofuran-d_6, 298 K) of 3,9-Br_2PXX.

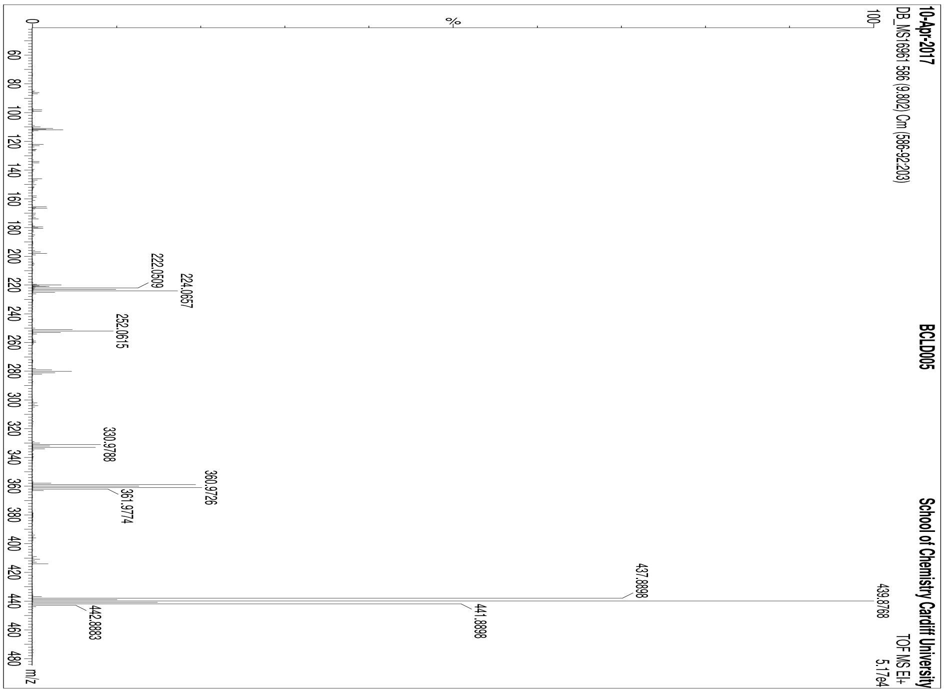

Figure S31. Mass spectra of 3,9-Br_2PXX.

Single Mass Analysis
Tolerance = 10.0 PPM / DBE: min = -1.5, max = 50.0
Element prediction: Off

Monoisotopic Mass, Odd and Even Electron Ions
7 formula(e) evaluated with 1 results within limits (all results (up to 1000) for each mass)
Elements Used:
C: 0-20 H: 0-8 O: 0-2 Br: 0-2

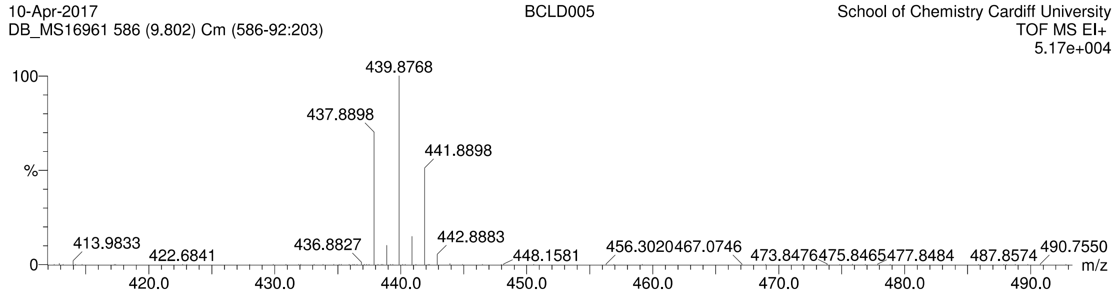

Minimum:				-1.5		
Maximum:		5.0	10.0	50.0		
Mass	Calc. Mass	mDa	PPM	DBE	i-FIT	Formula
437.8898	437.8891	0.7	1.6	16.0	1245.1	C20 H8 O2 Br2

Figure S32. Elemental composition report of 3,9-Br_2PXX.

X-ray analysis. Single crystals of 3,9-Br_2PXX were grown by slow evaporation of THF. A single crystal was mounted in Fomblin and diffraction data recorded at Diamond Light Source Beamline I19-1 using Synchrotron radiation (λ = 0.6889 Å) and a photon counting pixel array detector. Measurements were made at 100(2) K with the temperature maintained using an Oxford Cryostream apparatus. Data were collected, integrated and corrected for absorption by an empirical model using DIALS 1.dev.3189-g0030a24382[32] and XIA2 0.5.848-g17d2c5cf.[24] The structure was solved by direct methods (SHELXS)[25] and refined against F^2 within SHELXL.[26] A summary of crystallographic data are available as further supporting information and the structure has been deposited with the Cambridge Structural Database (CCDC deposition number 1901257). These data can be obtained free of charge from The Cambridge Crystallographic Data Centre via www.ccdc.cam.ac.uk/data_request/cif.

Table S4. Crystal data and structure refinement for 3,9-Br_2PXX.

Identification code	shelx
Empirical formula	C20 H8 Br2 O2
Formula weight	440.08
Temperature	100(2) K
Wavelength	0.6889 ≈
Crystal system	Triclinic
Space group	P -1
Unit cell dimensions	a = 3.8863(3)≈ a= 95.852(7)∞.
	b = 7.1449(6)≈ b= 95.766(6)∞.
	c = 13.1979(10) ≈ g = 96.410(6)∞.
Volume	359.95(5) ≈3
Z	1
Density (calculated)	2.030 Mg/m3
Absorption coefficient	5.213 mm-1
F(000)	214
Crystal size	0.111 x 0.026 x 0.010 mm3
Theta range for data collection	1.513 to 35.798∞.
Index ranges	-6<=h<=6, -11<=k<=11, -21<=l<=21
Reflections collected	7022
Independent reflections	3209 [R(int) = 0.0513]
Completeness to theta = 24.415∞	98.5 %
Absorption correction	Empirical
Max. and min. transmission	1.0 and 0.998883499336
Refinement method	Full-matrix least-squares on F2
Data / restraints / parameters	3209 / 0 / 109
Goodness-of-fit on F2	1.003
Final R indices [I>2sigma(I)]	R1 = 0.0456, wR2 = 0.1274
R indices (all data)	R1 = 0.0539, wR2 = 0.1317
Extinction coefficient	n/a
Largest diff. peak and hole	3.142 and -1.308 e.≈-3

Table S5. Atomic coordinates (x 104) and equivalent isotropic displacement parameters (≈^{2}x 103) for 3,9-Br_2PXX. U(eq) is defined as one third of the trace of the orthogonalized Uij tensor.

	x	y	z	U(eq)
C(1)	7376(6)	3594(4)	6557(2)	19(1)
C(2)	8421(7)	3159(4)	7524(2)	24(1)
C(3)	8040(7)	4459(4)	8390(2)	25(1)
C(4)	6638(6)	6104(4)	8261(2)	22(1)
C(5)	5456(6)	6582(4)	7276(2)	20(1)
C(6)	3909(6)	8244(4)	7073(2)	21(1)
C(7)	2818(6)	8593(4)	6089(2)	21(1)
C(8)	3267(6)	7285(4)	5255(2)	19(1)
C(9)	4762(6)	5669(3)	5420(2)	17(1)
C(10)	5863(6)	5289(4)	6420(2)	18(1)
O(1)	7870(5)	2341(3)	5727(1)	21(1)
Br(1)	6295(1)	7793(1)	9440(1)	28(1)

Table S6. Anisotropic displacement parameters (≈^{2}x 10^3)for 3,9-Br_2PXX. The anisotropic displacement factor exponent takes the form: -2p2[h^2a^{*2}U11 + ... + 2 h k a* b* U^{12}]

	U11	U22	U33	U23	U13	U12
C(1)	15(1)	28(1)	15(1)	6(1)	2(1)	2(1)
C(2)	22(1)	33(1)	18(1)	10(1)	0(1)	4(1)
C(3)	21(1)	38(1)	19(1)	9(1)	2(1)	4(1)
C(4)	16(1)	35(1)	15(1)	5(1)	2(1)	2(1)
C(5)	15(1)	30(1)	15(1)	3(1)	4(1)	2(1)
C(6)	18(1)	30(1)	17(1)	2(1)	3(1)	4(1)
C(7)	17(1)	27(1)	21(1)	4(1)	3(1)	4(1)
C(8)	15(1)	26(1)	17(1)	7(1)	2(1)	3(1)
C(9)	15(1)	24(1)	15(1)	5(1)	3(1)	3(1)
C(10)	13(1)	27(1)	13(1)	4(1)	2(1)	2(1)
O(1)	24(1)	26(1)	16(1)	6(1)	2(1)	7(1)
Br(1)	23(1)	48(1)	14(1)	-1(1)	1(1)	10(1)

Table S7. Bond lengths [≈] and angles [∞] for 3,9-Br_2PXX.

C(1)-C(2)	1.376(3)
C(1)-O(1)	1.385(3)
C(1)-C(10)	1.423(4)
C(2)-C(3)	1.428(4)
C(2)-H(2)	0.9500
C(3)-C(4)	1.368(4)
C(3)-H(3)	0.9500
C(4)-C(5)	1.422(3)
C(4)-Br(1)	1.895(3)
C(5)-C(10)	1.418(4)
C(5)-C(6)	1.427(4)
C(6)-C(7)	1.381(3)
C(6)-H(6)	0.9500
C(7)-C(8)	1.408(4)
C(7)-H(7)	0.9500
C(8)-C(9)	1.376(3)
C(8)-O(1)#1	1.388(3)
C(9)-C(10)	1.410(3)
C(9)-C(9)#1	1.428(5)
C(2)-C(1)-O(1)	117.9(2)
C(2)-C(1)-C(10)	120.7(2)
O(1)-C(1)-C(10)	121.4(2)
C(1)-C(2)-C(3)	118.9(3)
C(1)-C(2)-H(2)	120.5
C(3)-C(2)-H(2)	120.5
C(4)-C(3)-C(2)	120.5(2)
C(4)-C(3)-H(3)	119.7
C(2)-C(3)-H(3)	119.7
C(3)-C(4)-C(5)	122.2(2)
C(3)-C(4)-Br(1)	118.39(18)
C(5)-C(4)-Br(1)	119.4(2)
C(10)-C(5)-C(4)	116.9(2)
C(10)-C(5)-C(6)	117.2(2)
C(4)-C(5)-C(6)	125.9(2)
C(7)-C(6)-C(5)	122.1(2)
C(7)-C(6)-H(6)	119.0
C(5)-C(6)-H(6)	119.0
C(6)-C(7)-C(8)	119.4(2)
C(6)-C(7)-H(7)	120.3
C(8)-C(7)-H(7)	120.3
C(9)-C(8)-O(1)#1	121.4(2)
C(9)-C(8)-C(7)	120.2(2)
O(1)#1-C(8)-C(7)	118.4(2)
C(8)-C(9)-C(10)	121.0(2)
C(8)-C(9)-C(9)#1	120.8(3)
C(10)-C(9)-C(9)#1	118.2(3)
C(9)-C(10)-C(5)	120.1(2)
C(9)-C(10)-C(1)	119.1(2)
C(5)-C(10)-C(1)	120.7(2)
C(1)-O(1)-C(8)#1	119.0(2)

Symmetry transformations used to generate equivalent atoms:
#1 -x+1,-y+1,-z+1

References

1. Berdonces-Layunta, A. *et al.* Chemical stability of (3,1)-chiral graphene nanoribbons. *ACS Nano* **15**, 5610–5617 (2021)
2. Lawrence, J. *et al.* Circumventing the stability problems of graphene nanoribbon zigzag edges. *Nat. Chem.* **14**, 1451–1458 (2022)
3. Smerieri, M. *et al.* Synthesis of graphene nanoribbons with a defined mixed edge-site sequence by surface assisted polymerisation of (1,6)-dibromopyrene on Ag(110). *Nanoscale* **8**, 17843–17853 (2016)
4. Batra, A. *et al.* Probing the mechanism for graphene nanoribbon formation on gold surfaces through X-ray spectroscopy. *Chem. Sci.* **5**, 4419–4423 (2014)
5. Berdonces-Layunta, A. *et al.* Order from a mess: the growth of 5-armchair graphene nanoribbons. *ACS Nano* **15**, 16552–16561 (2021)
6. Cai, J. *et al.* Atomically precise bottom-up fabrication of graphene nanoribbons. *Nature* **466**, 470–473 (2010)
7. De Oteyza, D. G. *et al.* Substrate-independent growth of atomically precise chiral graphene nanoribbons. *ACS Nano* **10**, 9000–9008 (2016)
8. Kimouche, A. *et al.* Ultra-narrow metallic armchair graphene nanoribbons. *Nat. Commun.* **6**, 10177 (2015)
9. Lawrence, J. *et al.* Probing the magnetism of topological end states in 5-armchair graphene nanoribbons. *ACS Nano* (2020)
10. Schulz, F. *et al.* Precursor geometry determines the growth mechanism in graphene nanoribbons. *J. Phys. Chem. C* **121**, 2896–2904 (2017)
11. Lee, J., Kalin, A. J., Yuan, T., Al-Hashimi, M., Fang, L. Fully conjugated ladder polymers. *Chem. Sci.* **8**, 2503–2521 (2017)
12. Lawrence, J. *et al.* Combining high-resolution scanning tunnelling microscopy and first-principles simulations to identify halogen bonding. *Nat. Commun.* **11**, 2103 (2020)
13. Jeon, U. S. *et al.* Two-dimensional networks of brominated Y-shaped molecules on Au(111). *Appl. Surf. Sci.* **432**, 332–336 (2018)
14. Yoon, J. K. *et al.* Visualising halogen bonds in planar supramolecular systems. *J. Phys. Chem. C* **115**, 2297–2301 (2011)
15. Krejčí, O., Hapala, P., Ondráček, M., Jelínek, P. Principles and simulations of high-resolution STM imaging with a flexible tip apex. *Phys. Rev. B* **95**, 045407 (2017)
16. Atkins, P. W., Friedman, R. S. Molecular quantum mechanics, 4th edn., ch. 8 (Oxford University Press, 2010)
17. Becke, A. D. Density-functional thermochemistry. III. the role of exact exchange. *J. Chem. Phys.* **98**, 5648–5652 (1993)
18. Dion, M., Rydberg, H., Schröder, E., Langreth, D. C., Lundqvist, B. I. Van der Waals density functional for general geometries. *Phys. Rev. Lett.* **92**, 246401 (2004)
19. Krukau, A. V., Vydrov, O. A., Izmaylov, A. F., Scuseria, G. E. Influence of the exchange screening parameter on the performance of screened hybrid functionals. *J. Chem. Phys.* **125**, 224106 (2006)
20. Pummerer, R., Prell, E., Rieche, A. Darstellung von binaphthylendioxyd. *Ber. Dtsch. Chem. Ges.* **59**, 2159–2161 (1926)
21. Kobayashi, N., Sasaki, M., Nomoto, K. Stable peri-xanthenoxanthene thin-film transistors with efficient carrier injection. *Chem. Mater.* **21**, 552–556 (2009)
22. Pat. Appl.WO2008/011964A1 (2008)

23. Winter, G. xia2: an expert system for macromolecular crystallography data reduction. *J. Appl. Crystallogr.* **43**, 186–190 (2010)
24. Winter, G. *et al.* DIALS: implementation and evaluation of a new integration package. *Acta Crystallogr. D* **74**, 85–97 (2018)
25. Sheldrick, G. M. A short history of SHELX. *Acta Crystallogr. A* **64**, 112–122 (2008)
26. Sheldrick, G. M. Crystal structure refinement with SHELXL. *Acta Crystallogr. C* **71**, 3–8 (2015)
